# Evolving the Era of 5D Ultrasound? A Systematic Literature Review on the Applications for Artificial Intelligence Ultrasound Imaging in Obstetrics and Gynecology

**DOI:** 10.3390/jcm12216833

**Published:** 2023-10-29

**Authors:** Elena Jost, Philipp Kosian, Jorge Jimenez Cruz, Shadi Albarqouni, Ulrich Gembruch, Brigitte Strizek, Florian Recker

**Affiliations:** 1 Department of Obstetrics and Gynecology, University Hospital Bonn, Venusberg Campus 1, 53127 Bonn, Germany; 2 Department of Diagnostic and Interventional Radiology, University Hospital Bonn, Venusberg Campus 1, 53127 Bonn, Germany; 3 Helmholtz AI, Helmholtz Munich, Ingolstädter Landstraße 1, 85764 Neuherberg, Germany

**Keywords:** systematic review, ultrasound imaging, artificial intelligence, deep learning, obstetrics, gynecology, fetal echocardiography, application

## Abstract

Artificial intelligence (AI) has gained prominence in medical imaging, particularly in obstetrics and gynecology (OB/GYN), where ultrasound (US) is the preferred method. It is considered cost effective and easily accessible but is time consuming and hindered by the need for specialized training. To overcome these limitations, AI models have been proposed for automated plane acquisition, anatomical measurements, and pathology detection. This study aims to overview recent literature on AI applications in OB/GYN US imaging, highlighting their benefits and limitations. For the methodology, a systematic literature search was performed in the PubMed and Cochrane Library databases. Matching abstracts were screened based on the PICOS (Participants, Intervention or Exposure, Comparison, Outcome, Study type) scheme. Articles with full text copies were distributed to the sections of OB/GYN and their research topics. As a result, this review includes 189 articles published from 1994 to 2023. Among these, 148 focus on obstetrics and 41 on gynecology. AI-assisted US applications span fetal biometry, echocardiography, or neurosonography, as well as the identification of adnexal and breast masses, and assessment of the endometrium and pelvic floor. To conclude, the applications for AI-assisted US in OB/GYN are abundant, especially in the subspecialty of obstetrics. However, while most studies focus on common application fields such as fetal biometry, this review outlines emerging and still experimental fields to promote further research.

## 1. Introduction

Artificial intelligence (AI) is known to be present in everyday life, and over the past years it has gained considerable significance in medical imaging. The term AI refers to various types of computer science technologies, which enable machines to perform tasks simulating human intelligence. AI systems are typically dependent on the input of vast amounts of data, e.g., for pattern recognition, in order to learn to create predictions, classifications, recommendations, or decisions either supervised by humans or without supervision. Terms like machine learning or deep learning represent two of the numerous subcategories of AI [1].

Widely described advantages of AI usage include improved productivity, efficiency, and reduction in human error. These benefits are what make AI exceptionally attractive for application in health care and, particularly, in medical imaging [1]. To comply with the growing demand for AI software in health care, the U.S. Food and Drug Administration has currently certified a list of 178 AI-enabled medical devices, which is continuously growing [2].

The field of obstetrics and gynecology (OB/GYN) is known to be one of the most applied imaging specialties using the diagnostic tools of ultrasound (US), magnetic resonance imaging (MRI), computed tomography (CT), positron emission tomography (PET), laparoscopy, and others. In particular, the subspecialty of obstetrics is profoundly dependent on the diagnostic imaging tool of US because of its non-invasive, cost-effective, real-time, and low-radiation characteristics for fetal scanning [3,4].

However, US imaging has limitations regarding its comparability and reproducibility. Reasons for the high image variability can be low image quality, the need for real-time interpretation, differences in US devices, and the dependence on the sonographer’s experience [4]. The limitations increase when US is performed during pregnancy, facing obstacles such as imaging artefacts by maternal (e.g., thickened abdominal wall in obese patient) or fetal (e.g., fetal position or movements) factors, reduced tissue contrast (e.g., with reduced amniotic fluid), and characteristics of increasing gestational age (GA) (e.g., growing fetal volume and increasing ossification of bones) [4]. In clinical settings, US is known to be time consuming and can require a substantial amount of training and experience for specific indications, such as fetal echocardiography or neurosonography [5,6,7].

To overcome these restrictions, the application of AI assistance in US imaging has been shown to reduce examination time, clinician workload, and inter- and intra-observer variability [8]. US imaging in OB/GYN represents a promising field of application for AI models due to the wide range of indications and generation of high-volume data sets. Various operators with different skill levels and different US devices are challenging aspects influencing AI model performance.

However, the use of AI models is not without discussion about its ethical context [3]. Despite all efforts to automatize US imaging, existing literature still emphasizes the fact that AI is not meant to replace human work and input, but should assist them and reduce the increasing workload [3,6]. As stated in the state-of-the-art review by Drukker et al. [3], to date, no AI method exists that can be generalized to different tasks compared with an OB/GYN specialist capable of performing US scans of different organs and the fetus in various GA periods. Therefore, the variety of AI models in the subspecialties of OB/GYN is tremendous and is worth reviewing within the specific tasks.

So far, most original research articles and literature reviews in OB/GYN have focused on the common fields of AI application in medical imaging, such as the identification of breast or adnexal masses, automated fetal biometry, or fetal echocardiography. To the best of our knowledge, this review is the first to systematically display the variety of fields of applications among the subspecialties of OB/GYN.

The term ‘5D ultrasound’ is derived from the idea to expand the technological US world with a further dimension. While 4D technology extends the 3D view of the scanned object with a time frame, enabling motion visualization, or so-called ‘real-time 3D’ [9], the term 5D is uncommonly used to describe AI-assisted US imaging including image enhancement processing or automated calculations [10,11]. As there is no clear definition for 5D US, we use the expression of the ‘5D ultrasound’ in this literature review to illustrate the extend of AI models that can create a new dimension in US imaging in OB/GYN by improving work efficacy, accuracy, and visibility in clinical settings.

This study aims at providing an overview of the recent literature on applications for AI in US imaging in the medical field of OB/GYN by working out the benefits and limitations of AI US support systems. Special focus is given to the distribution of research attention among the subspecialties of OB/GYN and researching the emerging and still experimental fields to promote further research for clinical applicability. Assessing the effectiveness of applied AI models is not aim of this study; therefore, all AI technologies are summarized by the term ‘AI’. By describing the current research emphasis, possible missing scopes of application may be enlightened.

## 2. Materials and Methods

This systematic literature review was developed in accordance to the updated Preferred Reporting Items for Systematic Reviews and Meta-Analyses (PRISMA) statement [12,13]. The study was prospectively registered at the International prospective register of systematic reviews (PROSPERO) with registration number CRD42023434218.

For the literature research, the PubMed Database was searched on 14 May 2023 for records using the following search query and using the text availability filter ‘abstracts’:

*((artificial intelligence) OR (deep learning) OR (machine learning) OR (artificial neural networks)) AND (ultrasound) AND ((obstetrics) OR (gynecology) OR (pregnancy))*. Additionally, the Cochrane Library was searched on 14 May 2023 with the following query:

*(Artificial intelligence OR deep learning OR machine learning OR artificial neural networks) AND ultrasound AND (obstetrics OR gynecology OR pregnancy) [Title Abstract Keyword]*

No restriction for year of publication was applied. Relevant records in English or German were independently screened based on the title and abstract by two authors for their accordance with the eligibility criteria. Cases of incongruence were discussed in a consensus meeting. For adequate comprehensiveness of the search process, the PICOS search tool (Participants, Intervention or Exposure, Comparison, Outcome, Study type) was applied and used for judgement [12,13]. Table 1 shows the relevant literature characteristics presented as PICOS search tool headings. Records without the use of AI or US and studies focusing on AI applications in specialties other than OB/GYN were excluded in the screening process. Records describing AI calculations using data obtained from US measures but not the image itself (e.g., crown-rump-length (CRL) or cervical length) were excluded.

After the initial screening process, full text copies were retrieved for further analysis of the inclusion criteria. By extracting fields of applications, articles were distributed to the sections of either obstetrics or gynecology. By reading all full text copies, the topic of AI application (e.g., fetal neurosonography or identification of breast masses) and the specific benefits and limitations of the AI application in the presented field were extracted. The proportion of research topics for the included literature are illustrated in two figures for the subspecialties of OB/GYN.

## 3. Results

### 3.1. Included Literature

**Figure 1:**
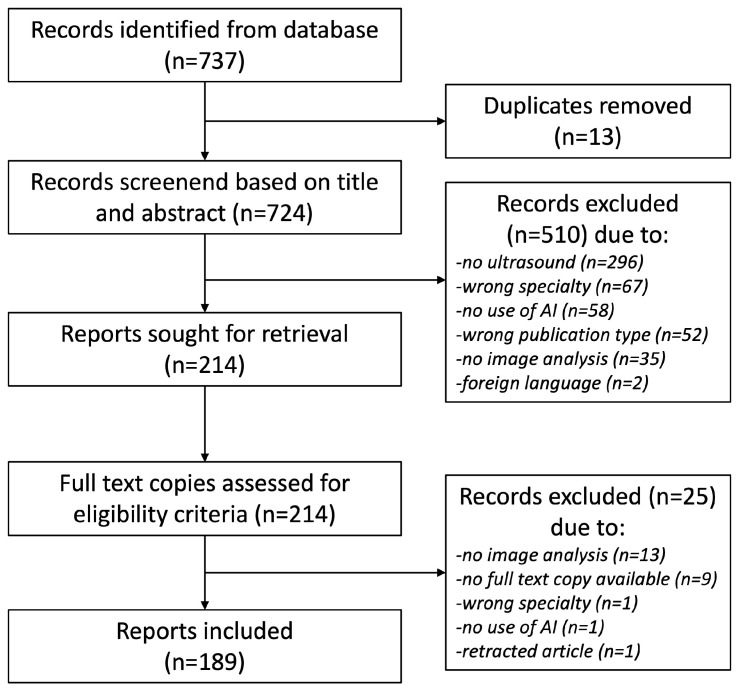
PRISMA flow diagram for the screening process of reports included in this review.

Figure 1 depicts the PRISMA flow diagram for the screening process of reports included in this review. A total of 737 records were identified from the searched databases, resulting in 189 records considered adequate for inclusion in this review. Here, 148 records described the application of AI in US imaging in the field of obstetrics compared with 41 records in the field of gynecology (Figure 1). The included articles are displayed in Table S1 of the Supplementary Material for obstetric and Table S2 of the Supplementary Material for gynecological applications. In the following Results section, included articles are evaluated separately for both specialties.

### 3.2. Applications in Obstetrics

**Figure 2:**
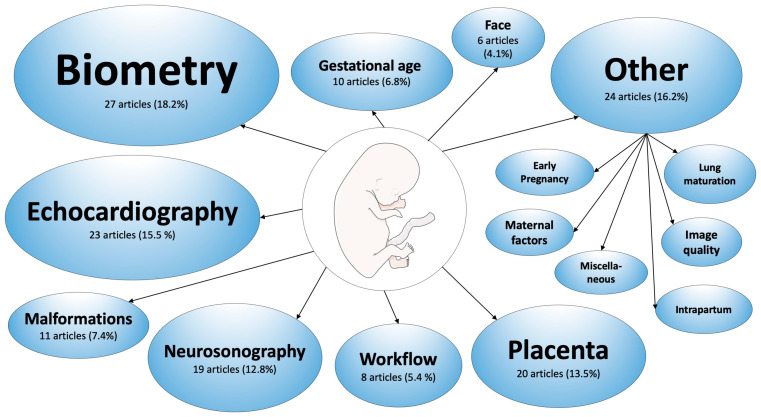
Overview of the distribution of research topics in the analyzed literature (a total of 148 articles) for AI applications in US imaging in the subspecialty of obstetrics. Figure adapted from Servier Medical Art.

Related to the subspecialty of obstetrics, 148 research articles form part of this systematic literature review. In Figure 2, an overview of the various research topics presented in the included literature is depicted.

#### 3.2.1. Fetal Biometry

The most common application of obstetric US examination remains the assessment of fetal growth, followed by the sonographic examination maternal−fetal perfusion parameters, fetal malformations, placental morphology, or uterine abnormalities. Fetal growth assessment represents a repeated standardized examination throughout pregnancy to monitor fetal development and predict birth weight, and consequently may influence decision making for the timing of delivery. Biometric calculations are based on the acquisition of standard planes for measurements of fetal head circumference (HC), abdominal circumference (AC), and femur length [14]. The accuracy of these measurements is often reduced as a result of operator-, equipment-, patient-, and fetus-related factors.

- Operator-related factors: US in general and obstetric US in particular, are known to require substantial experience and to be extremely training dependent [15]. US examinations are known to inherit high intra- and inter-operator variability [16].
- Equipment-related factors: Especially in hospital settings, repeated examinations may be performed with different US machines, resulting in heterogenous data. Furthermore, image quality depends on resource availability and access to high-end US devices [17], or the use of point-of-care devices [18].
- Patient-related factors: Maternal obesity is known to have an impact on image quality and visualization of the fetus, and thus limits the accuracy of obstetric US examinations [19].
- Fetus-related factors: Fetal size, movements, and position, as well as multiple pregnancies or reduced amniotic fluid resulting in low contrast to surroundings may decrease the accuracy of measurements [20,21].

To minimize operator- and/or equipment-related influences, recently, there have been attempts to automate measurements in obstetric US using AI algorithms. However, these attempts are characterized by their complexity and limitations due to the inevitable patient- and fetus-related constraints.

This review encompasses 27 research articles on the use of AI in the detection, measurement, and assessment of standard planes in obstetric US, with years of publication ranging from 2007 to 2023. Only three of the included studies investigated the use of AI algorithms in 3D US images [22,23,24], while 24 focused on 2D images. Most studies reported the combined analysis of various standard planes (13 studies, [15,17,23,24,25,26,27,28,29,30,31,32,33]) or exclusively presented an algorithm for the analysis of HC (9 studies, [18,22,34,35,36,37,38,39,40]), AC (4 studies, [21,41,42,43]) or femur length (1 study, [44]). Five studies used the freely available ‘HC18’ data set [45] for training and testing the algorithms, which contained 1334 2D images from 551 women of the standard fetal head plane [18,34,37,38,40].

AI algorithms for the automated detection of various standard planes in US video scans have been reported by Płotka et al. [25], Chen et al. [28], and Baumgartner et al. [15]. The unique study of Sendra-Balcells et al. presented a deep-learning model to identify standard planes in 2D images showing the transferability of the AI method to six low-income African countries [17]. In comparison with the analysis of 2D US videos, Sridar et al. [26], Burgos-Artizzu et al. [27], Rahman et al. [30], and Carneiro et al. [31,32] reported AI systems for the automated detection or measurement of various standard planes in 2D US images. Zhang et al. proposed an image quality assessment method for evaluating whether US images of standard planes fully show the anatomical structures with clear boundaries [29]. To improve clinical workflow efficiency, Luo et al. evaluated the intelligent Smart Fetus technique for its ‘one-touch’ approach to search and automatically measure the cine loop for standard planes once the sonographers press the freeze button [33]. Pluym et al. and Yang et al. analyzed 3D US volumes for the localization and measurement of various intracranial standard planes, such as the transventricular, transthalamic, or transcerebellar plane [23,24].

The automated detection and measurement of HC in 2D images was investigated by the research groups Zeng et al. [18,34], Li et al. [36,40], Yang et al. [37], and Zhang et al. [38]. Likewise, Van de Heuvel et al. and Arroyo et al. presented a system for automated analysis of HC, particularity the use of a standardized sweep protocol for data collection to eliminate the need for sonography experts and enable applicability in underserved areas [35,39]. The only study to identify the HC and biparietal diameter from 3D US volumes using the commercially available Smartplanes^®^ software was presented by Ambroise Grandjean et al. [22].

The acquisition and measurement of AC were considered in the studies of Jang et al. and Kim et al. analyzing 2D images [21,41], as well as by Ni et al. and Chen et al. analyzing 2D videos acquired by graduate students [42,43].

Remarkably, only one study, from Zhu et al., reported the automated assessment of femur lengths in 2D US images addressing the difficulties regarding femur lengths acquisition due to the complex background in femur US images [44].

In conclusion, the automated acquisition and measurement of standard planes is an increasingly investigated area, but still faces problems when it comes to clinical applicability and generalization. The benefits of AI usage in standard plane acquisition were found to be the possibility of real-time applicability [15,25,32,33]; the incorporation of clinical aspects into image interpretation [21,23,26]; the feasibility of biometric assessment by non-experts [35,39,43]; the application of a lightweight algorithm in point-of-care devices [18,34,37]; and, as a consequence of the latter two aspects, applicability for medically underserved areas [17,35].

The limitations of AI applications for fetal biometry were found to be reduced algorithm accuracy in poor quality images due to high maternal BMI [22,23], low contrast of anatomical structures [36], and higher GA with large fetuses [21]. Other reported limitations were slow processing times [35,43] and the lack of training for algorithms with pathological cases [28,35].

#### 3.2.2. Fetal Echocardiography

For the detection of the most common congenital malformations, which are known to be congenital heart diseases (CHDs), with an incidence of 6–12/1000 livebirths [46], fetal sonographic examination is usually performed in the second trimester [47,48]. The prenatal diagnosis of CHD is of substantial significance, resulting in improved neonatal outcomes compared with postnatal diagnosis. It allows for appropriate counseling for parents, as well as delivery and treatment planning, and, in some cases, even in utero therapy [49].

Fetal echocardiography is a highly challenging technique, even for experts, and is primarily based on the acquisition of standard views, such as the four-chamber view (4CV), three-vessel view, three-vessel trachea view, and left and right ventricular outflow tract view. The combination of standard views allows for the detection of up to 90% of CHD; however, in clinical practice, the detection rate is only about 30% [48,50]. Reasons for low detection rates were described as insufficient sonographer interpretation and inadequate acquisition of standard views, which were often due to fetus-related factors such as fetal position, movements, and the small size of the fetal heart and its possible defects [50].

This review includes a total of 23 articles from 2007 to 2023 related to fetal echocardiography. Four of the included studies investigated the application of fetal intelligent navigation echocardiography (FINE) as a reliable technique that enabled the automated acquisition of nine standard echocardiographic views from specific 4D volumes of a single cardiac cycle in motion [51,52,53,54]. It enabled operator-independent examination and contributed to the standardization of fetal echocardiography [52]. While Yeo et al. reported the time-saving benefits of workflows using FINE in 51 normal fetal cardiac anatomy and 4 different CHD cases [51], Ma et al. confirmed the application of FINE in abnormal anatomical hearts through the successful generation of three standard views in 30 fetuses with double-outlet right ventricles [53]. In a case report, Veronese et al. reported the successful detection of four atrioventricular septum defects using the FINE system [54].

Five of the included studies investigated the performance of AI models that can detect structural abnormalities in cardiac anatomy. Dozen et al. presented a method specific to the interventricular septum [55]; Han et al. focused on the assessment of the left ventricle and left atrium [56]; and Xu et al. aimed at identifying seven anatomical structures, namely right and left atrium and ventricle, thorax, descending aorta, and epicardium [57]. The automated detection of standard views was investigated by Wu et al., Yang et al., and Nurmaini et al. [58,59,60], while CHD was effectively detected by AI models generated by the research groups of Gong et al. and Nurmaini et al. [61,62], and selectively for the diagnosis of total anomalous pulmonary venous connection by Wang et al. [63].

Three recent studies assessed fetal cardiac function. Yu et al. automatically measured left ventricular volume in 2D US images [64], Herling et al. analyzed the automated measurement of fetal atrioventricular plane displacement in US videos of cardiac cycles using color tissue Doppler [65], and Scharf et al. evaluated the automated assessment of the myocardial performance index as a tool to analyze fetal cardiac function [66]. Lastly, two included studies developed AI models for model improvement itself by synthesizing high-quality 4CV images for model training [67] and by providing existing models with new input data and supporting learning process [68].

The complexity of fetal echocardiography itself is derived from the skill needed to detect even the smallest anatomical abnormalities in a beating organ, which makes it an interesting and challenging research area for AI applications. Advantages are the facilitation of standard view acquisition [58,60] and CHD detection [53,61,62], as well as a significant reduction in examination time [52,55]. Furthermore, Arnaout et al. outlined the benefits of their AI model for telehealth approaches and diagnoses of rare diseases [50]. In case of the study of Emery et al., an AI-based navigation system for needle tracking in fetal aortic valvuloplasty promised increased safety, reduced intervention time, and transferability for other fetal interventions such as amniocentesis [69].

As a result of AI models acquiring US images, the need for quality control mechanisms has arisen to ensure image quality. This issue was addressed by Dong et al. and Pietrolucci et al., who developed quality assessment AI models, of which one is already commercially available, known as ‘Heartassist™’ [70,71]. Furthermore, to address the ‘black box problem’, which describes the complexity of algorithms impossible for human understanding, Sakai et al. proposed a method to support fetal echocardiography through ‘explainable AI’ [7]. This technique aims at promoting the trustworthy use of AI methods for clinicians through the development of specific AI modules for the explanation of the algorithm behavior.

AI assistance in fetal echocardiography showed several limitations. First, most of the studies only used 4CV images as the input for their algorithms [55,57,61,62,63,67,70], although the detection rate of CHD could be increased by analyzing different standard views [55]. These studies predominantly used apical 4CV, which resulted in AI model limitations when analyzing 4CV from different scanning angles, such as the fetal dorso-anterior position. This issue of the need for the correct identification of the region of interest (ROI) for optimized AI model performance was addressed by the study of Xu et al. [57]. Second, analyzed images have often been obtained only from healthy fetuses with normal cardiac anatomy and AI models lacked training with pathologic findings [52,55,57,64]. Furthermore, even with the assistance of AI methods, experienced sonographers were required for rechecking and the interpretation of results [7,51]. Lastly, the recognition of small CHD or small anatomical structures such as the trachea was limited in some models [55,58].

In summary, fetal echocardiography extensively profits from AI assistance, but shows limitations that need to be addressed in further research. Beside the aforementioned need for the automated detection of ROI, which has been recently proposed in the literature [72,73], other fields of application are of emerging interest. Not only the detection of structural abnormalities in case of CHD, but also cardiac function analysis, is a future topic of AI applications in fetal echocardiography using tissue Doppler US, which can be relevant, e.g., for fetuses diagnosed with hypoplastic left heart syndrome [74,75,76].

#### 3.2.3. Fetal Neurosonography

Fetal neurosonography focuses on the assessment of fetal brain development and the identification of abnormalities [77]. For sonographic assessment, standard head planes should be acquired following the international guideline of the International Society of Ultrasound in Obstetrics and Gynecology (ISUOG) [77], which enables the detection of key anatomical structures such as lateral ventricles, cavum septum pellucidum, cerebellum, and cisterna magna. Sonographers performing neurosonography require an accurate understanding of fetal neuroanatomy, the skill to interpret 2D planes in a complex 3D structure, and, consequently, substantial clinical experience and training [78].

On the topic of neurosonography, 19 studies were included in this review, ranging from 2017 to 2022 in years of publication. Almost half of the included studies investigated AI applications in US using 3D volumes. Three of the included studies [78,79,80] used data collected in the Fetal Growth Longitudinal Study of the INTERGROWTH-21st Project, which aimed at developing international standards in fetal growth and size [81].

The research topics in this field are heterogenous, presenting a wide variety of applications for AI-assisted methods. The establishment of a plane localization system as a 3D reference space for locating 2D planes was proposed by Yeung et al., Namburete et al., Yu et al., and Di Vece et al. for improving the acquisition of standard planes and facilitating anatomical orientation for sonographers [78,80,82,83]. In particular, the method by Di Vece et al. used a 23-week synthetic fetal phantom for system development and was the only study to estimate the 6D poses of US planes combining common 3D planes with rotation around the brain center [82]. Xu et al. presented an AI method for authentically simulating third-trimester images from second-trimester images for deep-learning researchers with restricted access to third-trimester images [84]. The automated detection of brain structures and malformations was described by Lin et al. [85,86], Alansary et al. [87], and Gofer et al. [88] in 2D images and videos, and in 3D volumes by Hesse et al. and Huang et al. [79,89]. The image quality assessment of whether a standard plane was correctly acquired, either by human operators or by automated extraction from 3D images, was effectively performed by models developed by the research groups of Lin et al. [90], Yaqub et al. [91], and Skelton et al. [92]. Researchers Xie et al. [6,93] and Sahli et al. [94] reported a method for classifying US images into a binary system of ‘normal’ and ‘abnormal’ cases, in which Xie et al. additionally localized the structural lesions, which lead the algorithm to declare it ‘abnormal’ and thus recommend the clinician to recheck the labeled area. Lastly, the studies of Burgos-Artizzu et al. and Sreelakshmy et al. portrayed AI methods for the estimation of GA through an analysis of transthalamic axial planes or cerebellum measurements [95,96].

The benefits of the usage of AI algorithms in fetal neurosonography were, beside a reduced workload for sonographers due to faster acquisition and measurements [86], the development of guiding methods for skill training [78,83], the measurement of small anatomical structures such as the fetal cortex in first trimester [88], and the accurate estimation of GA in a pregnancy without a valid first trimester scan [95].

The primary limitation of AI US imaging in this topic was described to be the rapid anatomical development of fetal brain structures due to brain maturation, increasing head size and degree of ossification with rising GA [78,80,84]. Ossification of the fetal skull provoked an increase in the shadowing of US images and thus reduced image quality and visibility [80]. To address the heterogeneity in brain images from different GA, studies described the need for matching GA of US images in algorithms [82,94]. Other study limitations were the missing training of AI algorithms with images of pathologies [79,86,95] and the problem of miscalculations when US images were not in accordance with the guidelines for standard planes [6,80].

#### 3.2.4. Fetal Face

With advances in obstetric US and the possibility of 3D and 4D US, the analysis of the fetal face has become feasible and of rising interest. This section encompasses five articles from 2018 to 2023, with heterogenous research topics.

Fetal facial malformations, such as cleft lip and palate, can be assessed by acquiring standard planes such as the ocular axial, median sagittal, and nasolabial coronal plane [97]. Wang et al. and Yu et al. presented AI algorithms to automatically identify standard planes in 2D images [97,98]. However, as facial malformations can be a phenotype of an underlying genetic disorder, Tang et al. used 3D images of fetal faces to develop a novel approach for the early, non-invasive identification of genetic disorders by analyzing key facial regions, such as the jaw, frontal bone, and nasal bone [99].

Additionally, fetal movements and facial expressions were found to be correlated with fetal brain activity and development state [100]. Facial expressions such as eye blinking, mouthing, smiling, and yawning have been described to indicate fetal brain maturation, in utero stress may result in scowling, while the meaning of tongue expulsion and neutral expression remain unclear [101,102]. Miyagi et al. proposed an AI classifier analyzing 4D US volumes to assess fetal facial expressions and classify them into different categories [102,103], and showed that the identification of dense and sparse states of brain activity is possible [104].

#### 3.2.5. Placenta and Umbilical Cord

The placenta is known to play an important role in the pathogenesis of obstetric complications such as placenta previa, abnormally invasive placenta, fetal growth restriction, and hypertensive disorders of pregnancy [105]. Little evidence exists on the neglected role of the placental characteristics and the prediction of these complications [106]. For example, research has shown a correlation between early placental sonographic echogenicity and the prediction of intrauterine growth restriction [107]. To date, sonographic placental assessment is mainly restricted to the identification of placental location, adhesion, or insertion site of umbilical cord [108], and further assessment is limited due to the impossibility to detect minimal change in texture by routine scan and time-consuming examinations. The use of AI imaging algorithms has recently enabled automated assessment of the placental volume, tissue texture, and vascularization, and is thus of rising research interest.

In this review, 20 articles were included that were published from 1994 to 2023, whereby the three studies from 1994–1996 investigated umbilical cord Doppler analysis and studies from 2014–2023 focused on placental analysis. While part of the included studies focused on the automated assessment of placental localization and volume, others reported efforts to identify or predict the presence of placenta-related obstetric complications by analyzing echogenic tissue texture.

Andreasen et al. and Schilpzand et al. presented an effective AI algorithm for placental localization, including heterogenous data through differences in sonographers’ expertise [109], or using a previous established sweep protocol in low-resource settings [110]. It is known that early reduced placental volume is associated with small-for-gestational-age fetuses [111]. Schwartz et al. and Looney et al. presented an effective model to automatically assess placenta volumes from 2D and 3D images in first trimester [112,113]. Hu et al. performed an echotexture analysis in 2D placental images [108], while Qi et al. reported successful automated localization of the placental lacunae in 2D images as a potential tool for screening abnormally invasive placenta [114,115]. The automated classification of placental maturity was proposed by Lei et al. and Li et al. [116,117]. Early and small changes in placental tissue texture were detected by Gupta et al. and Sun et al. through using AI-assisted US and microvascular Doppler imaging in women with hypertensive disorders of pregnancy [118,119] and gestational diabetes [120].

Further examples of adverse pregnancy events with an often disastrous maternal and fetal outcome are placental abruption and pernicious placenta previa. Yang et al., therefore, investigated the predictive role of a scoring system for the occurrence of pernicious placenta previa [121], while the research group of Asadpour et al. reported a method for identifying placental abruption [122].

In addition to placental characteristics, research exists on the role of umbilical cord anatomy and blood flow. Pradipta et al. investigated the use of machine learning methods to classify 2D color Doppler US images of umbilical cords along the umbilical coiling index and its possible impact on fetal growth [123]. In the earliest studies included in this review, Beksaç et al. and Baykal et al. presented an automated diagnostic, interpretation, and classification method to analyze umbilical artery blood flow Doppler US images [124,125,126].

The importance of pre-operative planning for surgical interventions such as laser-therapy in twin-to-twin-transfusion syndrome was addressed by the research group of Torrents-Barrena et al. [127,128]. They proposed a new AI algorithm for the simulation and planning of fetoscopic surgery through the detection and mapping of the maternal soft tissue, uterus, placenta, and umbilical cord via MRI in combination with the detection of the placenta and its vascular tree in 3D US. This model fully simulates the intraabdominal environment and enables the correct entry point planning and surgeon’s training [127,128].

In summary, placental AI-based US diagnostic may propose a promising non-invasive, predictive tool to improve patient counseling and management to prevent adverse pregnancy outcomes. Reported limitations in applications arose from the difficulty of identifying the interface between the placenta and myometrium, especially in first trimester scans [113], and low accuracy rates in the assessment of posterior wall placentas [109]. Further research is necessary to identify the link between placental health and obstetric complications.

#### 3.2.6. Fetal Malformations

##### First Trimester Scan

The timing of the first trimester scan is standardized to 11 + 0 and 13 + 6 weeks of gestation and its performance of image acquisition is defined by a protocol of the Fetal Medicine Foundation [129] and the ISUOG [130]. The purpose of this US examination includes confirmation of viability; assessment of GA; screening for preeclampsia; and detecting chromosomal anomalies such as trisomy 13, 18, or 21 or other malformations. Combining clinical information (maternal age and serum parameters) with sonographic assessment of fetal characteristics, predominantly the assessment of nuchal translucency (NT), is recommended practice [131].

Seven studies included in this review focused on the AI application for first trimester scans, ranging from 2012 to 2022. The research groups Walker et al. [132], Zhang et al. [133], Sciortino et al. [134], and Deng et al. [135] addressed the time consuming process of NT measurement by introducing AI models for its automated detection and measurement, in particular for the diagnosis of trisomy 21 [133] or cystic hygroma [132]. Tsai et al. aimed at facilitating the preliminary step for NT measurement, which was the automated detection of the correct mid-sagittal plane in 3D volumes [136], and Ryou et al. and Yang et al. proposed a model for the assessment of the whole fetus in 3D volumes [137,138]. The potential benefits of these models were the highly accurate non-invasive method for anomaly screening [134] and reduced workload [133,135,136,137]. Limitations could be uncovered when assessing fetal limbs due to its small anatomy and close surroundings [136,137,138], small data sets in rare anomalies [132,133], and missing real-time application for clinical applicability [133,134,138].

##### Second Trimester Scan

The timing of the second trimester scan is standardized to 18 + 0 to 23 + 6 weeks of gestation and is intended for the evaluation of fetal growth and detection of fetal malformations [139].

Four studies included in this review focussed on the detection of fetal malformations in mid-trimester US scans. Matthew et al. prospectively evaluated a model for automated image acquisition, measurements, and report production [140]. Cengizler et al. proposed an algorithm for the identification of the fetal spine and proofed the model’s performance in cases of fetuses with spina bifida [141]. Furthermore, Meenakshi et al. focused on the identification of fetal kidneys [142] and Shozu et al. presented an AI model for the identification of the thoracic wall, which enabled plane detection for 4CV, but also allowed for the detection of thoracic malformations [143]. All of the studies showed a reduction in examination time, which helps sonographers concentrate on interpretation instead of repetitive tasks [140].

#### 3.2.7. Prediction of Gestational Age

The estimation of GA is one of the important indications for obstetric US in early pregnancy that helps to adjust maternity care and identify complications such as prematurity or fetal growth disorders [144]. It is usually calculated using the last menstrual period and is confirmed with fetal CRL and biometry. In low-resource countries, access to medical care is constrained and ultrasonography and their operators are rare. In these areas especially, pregnancy complications play an important role and improvement in diagnostic resources for correct GA measurement as a prerequisite for adequate maternity care is thus necessary [145].

This literature review includes 10 studies on the assessment of GA, starting in 1996 with a pioneering study of Beksaç et al. on the estimation of GA via the calculation of the fetal biparietal diameter and HC [146]. In addition to this, the studies of Namburete et al. and Alzubaidi et al. similarly used the anatomy and growth of the fetal head for GA estimation [147,148]. Dan et al. developed a DeepGA model that used the three main factors of fetal head, abdomen, and femur [149], while Lee et al. proposed a machine learning method to accurately estimate GA with standard US planes [150]. The recent topic of point-of-care-US was addressed in the research of Maraci et al., who successfully showed automated head plane detection and GA estimation with point-of-care devices [151]. Lastly, four studies used data from the Fetal Age Machine Learning Initiative (FAMLI), which is an obstetrical US development project in low-income settings. The purpose of these studies, which were based on US data from the US and Zambia, was the successful establishment of an AI algorithm for GA estimation from simplified blind US sweeps of US novices in low-resource countries [144,145,152,153]. The benefits of the GA AI models were the possibility for application in low-resource countries [144,145,148,149,152], even without internet connectivity [145], and in portable devices [148], promising high accuracy with an error of 3.9 to 5 days in GA estimation [149,152]. An important limitation of the AI models was described to be application in very early [144,147] or very late stages of pregnancy [146,147,152], the latter of which was due to the thickened texture of the fetal skull.

#### 3.2.8. Workflow Analysis of Obstetric Ultrasound Scans

Over the past decades, obstetrics US has gained immense advances in US technology and computational power, including AI processes, but the procedure of acquiring the image itself by a bedside-acting clinician has remained unchanged. As it is known that acquiring obstetric US skills is a long-lasting and highly demanding task, efforts have been made to analyze the workflow of experienced sonographers and draw conclusions about the interaction between the sonographer, probe, and image [154].

All eight included studies investigating this topic arose from the same working group of the University of Oxford, UK. The PULSE (Perception Ultrasound by Learning Sonographer Experience) project, presented by Drukker et al., was designed to enable insights into experts’ sonography workflow and to transform the learning process of obstetric US using deep-learning algorithms [154]. Its data set was within the framework of all of the included studies. While Drukker et al. analyzed eye and transducer movements, actions during scanning, and audio recordings to generate automated image captioning of the sonographer’s explanation, Sharma et al. added the pupillometric data to objectify not only the localization of the sonographer’s gaze on the screen, but also the intensity of concentration in this focus [155]. Completeness, precision, and speed of sonographic performance were assessed by Wang et al. to quantify operator skill level [156]. Zhao et al. presented a method for virtual-assisted probe movement guidance along a virtual 3D fetus model with automatic labeling of the captured images [157]. Sharma et al. and Drukker et al. analyzed full-length scanning videos to assess workflow by building timeline models illustrating the scanning sequence of anatomical regions over time [155,158]. Lastly, Alsharid et al. developed a novel video captioning model for the description of second-trimester US scans by training an AI model with speech recordings and gaze-tracking information of sonographers while performing US scans [159].

To sum up, the included studies on this topic showed clinical benefits of AI in scan workflow through automating image labeling [154,157], enabling transfer learning for US novices [160], reducing clinician’s mental workload, and optimizing workflow [155]. Limitations in application were reported to be the impossibility of the generalization of workflow sequences due to maternal−fetal factors and different skill levels of sonographers for different anatomical regions during a full routine scan [155,158].

#### 3.2.9. Other Applications in Obstetrics

##### Fetal Lung Maturation

The maturation process of the fetal lung is an important aspect in clinical practice as it implies the leading cause for neonatal morbidity and mortality [161]. The GA of the fetal lung does not always correlate with the actual GA and can be influenced by pregnancy complications disrupting lung maturation.

Five included studies analyzed fetal lung US images for the prediction of neonatal outcomes. Du et al. proposed an AI model to classify the lung textures in pregnancies affected by gestational diabetes or preeclampsia [162], while Xia et al. and Chen et al. developed a lung maturation grading model that can be implemented for identifying abnormal development and evaluating the effectiveness of antenatal corticosteroid therapy [161,163]. The study of Bonet-Carne et al. and a further study of Du et al. showed that automated fetal lung ultrasound was able to accurately predict neonatal respiratory morbidity [164,165]. While Du et al. analyzed lung images from healthy and affected pregnant women, the studies of Xia et al. and Chen et al. were limited by the analysis of only healthy fetuses [161,163].

##### Maternal Factors

US in OB/GYN usually focusses on the examination of the fetus; however, there are several indications to evaluate maternal structures.

Four included studies were summarized in this section. The early study of Wu et al. proposed a tool for preterm labor prediction by using computer-assisted measurement of the cervix on transvaginal US images to overcome the issues of poor reproducibility and sonographer dependency on manual cervical length measurements [166]. The model of He et al. addressed the challenge of the identification and classification of intrauterine pregnancy residues that had the potential to reduce associated complications and improve surgical outcomes of curettages [167]. Wang et al. presented an algorithm to assess the color Doppler US images of fetal and maternal vessels as an approach to facilitate the diagnosis of severe preeclampsia linked to the medical outcome [168]. Lastly, Liu et al. proposed a Doppler US model for the prediction of fetal distress in women with pregnancy-induced hypertension [169]. These algorithms may improve medical diagnosis and potentially reduce clinical workload by replacing the sonographer’s manual tracing [168].

##### Early Pregnancy

Three studies included in this review focused on the US examination in early pregnancy, which is often performed to confirm intrauterine localization and vitality of pregnancy, to estimate GA via measuring CRL, or to diagnose adverse pregnancy outcomes such as miscarriages.

While Wang et al. prospectively analyzed an automated assessment of the gestational sac as a predictor for early miscarriages in 2D images in pregnancies of 6–8 weeks of gestation [170], the research groups Sur et al. and Looney et al. proposed an 3D AI model to provide volumetric measurements of the embryo, placenta, gestational sac, yolk sac, and amniotic fluid [113,171]. The potential benefits of these models were the development of fetal volume nomograms for precise fetal growth assessment [171] and the establishment of an early screening method for the prediction of adverse pregnancy outcomes [113], which facilitates adequate consultancy and recommendation for follow-up US examinations [170]. However, validation of the results is difficult in this field of application, which limits clinical applicability.

##### Intrapartum Sonography

The application of US during the second stage of labor to assess the progression of childbirth is a recent development to improve obstetric management. Transperineal US is, therefore, used to objectivate vaginal digital examination when estimating fetal head descent.

In a prospective, multicenter study, Ghi et al. established an AI model for automatically classifying fetal head position and distinguishing between occiput anterior and non-occiput anterior position of the fetal head, because the latter may result in protracted labor and increased risk of a poor obstetric outcome [172]. In addition to this, Lu et al. and Bai et al. proposed a method for the automated measurement of the angle of progression, which allowed for the estimation of fetal head descent by identifying the symphysis and fetal head contour [173,174]. All three studies showed promising results but lacked clinical applicability for their missing real-time application.

##### Image Quality

In terms of the quality control of US images, four studies were included in this review that applied their algorithms to different aspects.

Wu et al. proposed a computerized quality assessment scheme for quality control of US images by identifying the ROI in fetal abdominal images [175]. Meng et al. established a model for the classification of shadow-rich and shadow-free regions in various US images [176] and Gupta et al. presented an algorithm for a better separation between the fetus and surrounding information in fetal US, such as maternal tissue, placenta, or amniotic fluid [177]. Lastly, Yin et al. showed improved image quality when using an AI algorithm for image processing in US images of the pelvic floor [178]. Beside improved US image quality [176,177,178], the benefits of automated quality control algorithms are the facilitation of image acquisition by novices and experts, reduced workload, and the development of toolkits for education [175].

##### Miscellaneous

Five studies investigating various areas are summarized in this section.

The study of Cho et al. proposed a model for the automated estimation of amniotic fluid, as it is known to be a particular observer-dependent factor and, therefore, benefits from automation [179]. Compagnone et al. presented a clinical case report of a successful AI-image-guided placement of an epidural catheter in an extremely obese patient for delivery [180]. The research group Maraci et al. developed an AI model to detect the fetal position and heart beat from predefined US sweeps. Further, Rueda et al. aimed at investigating the fetal nutritional status using AI-assisted assessment of the adipose and fat-free tissue of the fetal arm in US images [181]. Lastly, an AI model for the automated classification of fetal sex in 2D US images of the genital area was established by Kaplan et al. that helped reduce misclassification and facilitate screening [182].

### 3.3. Applications in Gynecology

**Figure 3:**
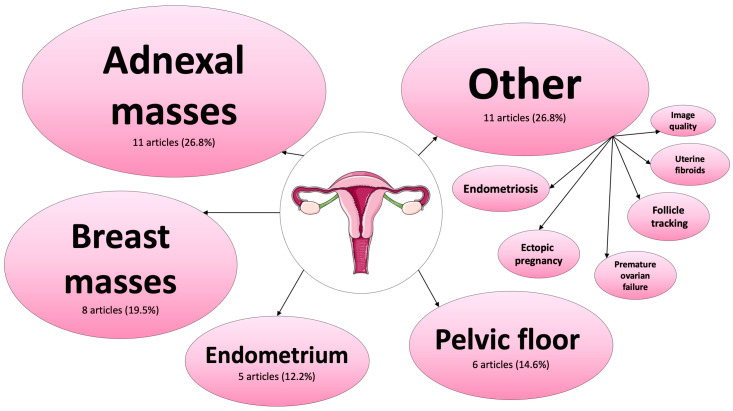
Overview of the distribution of research topics in the analyzed literature (a total of 41 articles) for AI applications in US imaging in the subspecialty of gynecology. Figure adapted from Servier Medical Art.

Focusing on the specialty of gynecology, 41 research articles were included in this review. Figure 3 provides an overview of research topics on AI applications in gynecological US.

#### 3.3.1. Adnexal Masses

Adnexal masses are among the common reasons for US examination in gynecology due to the importance of ovarian cancer detection. The assessment of adnexal findings is crucial for further diagnostic steps and therapy planning, which differ significantly between benign and malignant tumors. In the clinical setting, the examination of adnexal masses is primarily performed via transvaginal US, combining grayscale 2D images with color Doppler imaging to assess vascularization. The identification and especially classification of adnexal findings represents a challenging task even for experienced examiners and thus the International Ovarian Tumor Analysis (IOTA) group has established US-based rules for classification of adnexal tumors [183]. In recent years, the automated analysis of US images of adnexal masses has gained attention due to its advantage in supporting unexperienced examiners and assisting experienced examiners in diagnostic decision making.

Of the 11 extracted articles, only two were designed as prospective studies [184,185]. Included studies were published from 2009–2023, whereby the research group Amor et al. was the first to describe AI application in sonographic assessment using a non-specified pattern recognition analysis to classify adnexal masses in a new reporting system [184]. All but one of the studies analyzed 2D images, with only three of them including color Doppler images.

Enabling an automated discrimination between benign and malignant tumors was a predominant focus of the current research, represented in six studies included [185,186,187,188,189,190]. Three studies assessed the performance of automated tumor classification [184,191,192], one study developed a population-based screening method for BRCA mutations [193], and one study focused on the automated elimination of artefacts and objects in US images to increase the accuracy of the AI model [194]. Aramendía-Vidaurreta et al. was the only group to investigate the automated discrimination of benign and malignant masses in 3D US images [187] and Hsu et al. distinguished between transabdominal and transvaginal US images [185].

All of the included studies showed a high accuracy and sensitivity of AI performance. The study by Gao et al. used a large, multicenter, and heterogenous data set, which disclosed that AI-enabled US outperformed an average trained radiologist in discriminating malignant and benign ovarian masses and improved the examiner’s accuracy [189]. These findings were consistent with other studies [185,186], but there were also studies with smaller sample sizes that showed a level of performance reaching those of human experts [188,191,192].

Nevertheless, a described limiting aspect was the fact that clinicians using AI image analyzing algorithms must still take clinical aspects into account [188,189]. Furthermore, metastases or secondary ovarian cancer in pelvic images may be misinterpreted because of their different clinical presentation and their low representation in the data set [189]. Frequent described limitations of studies on AI applications in US imaging were homogeneity of data due to a single examiner or single center study [188,192], a single investigated ethnicity [189], absent external validation [192,193], poor image quality [186,190], and, most importantly, small sample sizes not sufficient enough to train the algorithm [184,185,187,188,192,193].

#### 3.3.2. Breast Masses

Breast cancer represents the most common malignancy in women worldwide and its incidence still shows a rising tendency [195]. To address this health issue, screening programs and early diagnosis are of the utmost importance. While primary screening is often performed and recommend through mammography, the advantages of breast US are numerous. Especially in women with dense breast tissue, e.g., predominantly in young women or in Asian ethnicity, and for underserved areas, US diagnostics and screening are crucial [196].

This review includes eight articles on AI application in US imaging of the breast, all of which were published in the past three years and focused on 2D images.

All of the included studies worked on either the detection of breast lesions, classification, or both. Two studies used AI algorithms in combination with handheld US devices [197,198]. Berg et al. pointed out the importance of training for sonographers to obtain a reasonable image quality for AI analysis [197], while Huang et al. compared handheld US to robotically performed AI-assisted US and showed reduced costs, shorter examination times, and a higher detection rate in the latter [198]. The possibility of avoiding unnecessary breast biopsies was the result of two further studies, of which one used an AI-assisted multi-modal shear wave elastography model [199,200]. In a retrospective study, Dong et al. promoted the importance of an increased confidence in AI assistance in health care, which can be addressed by understanding the algorithm of the black box and encouraging the concept of ‘explainable AI’ [201]. Limitations to AI usage in breast US were the missing clinical context in unimodal approaches only focusing on image analysis [202], small data sets for algorithm training, and a lower accuracy in borderline findings [201].

#### 3.3.3. Endometrium

In gynecologic US examinations, evaluation of the endometrium is part of normal routine and obtains its significance due to the frequency of endometrial abnormalities, e.g., endometrial fibroids, polyps, endometrial hyperplasia or atrophy, and carcinoma [203]. In particular, endometrial thickness is known to show dynamics in premenopausal women throughout the menstrual cycle, while an increase in thickness in postmenopausal women represents a risk factor for the presence of malignancy [204]. However, the identification of the endometrial−myometrial junction represents a challenging task due to heterogenous textures, irregular boundaries, and different sizes of the endometrium in the menstrual phases, which is why the application of AI in US is a field of research interest.

For this topic, five articles were extracted from the current literature. Publication years ranged from 2019 to 2023. Wang et al. and Zhao et al. conducted their studies based on 3D US images [205,206]. All but one study investigated the AI performance for the assessment of endometrial thickness, texture, or uterine adhesions [205,206,207,208]. Moro et al. aimed at establishing an AI model for risk stratification in endometrial cancer, but could not prove increased performance [209].

The application of AI US to assess endometrial characteristics showed a high accuracy and similar level of performance compared with human examiners [205,208], which could be further increased by setting human-selected key points in the images as a demarcation of the ROI for the AI algorithm [207]. The only two studies using 3D imaging outlined the superiority of this data to 2D imaging for its improved capability in identifying the endometrial−myometrial junction [205,206]. Extracted limiting aspects for AI application were reduced accuracy in assessing endometria smaller than 3 mm [208], operator-dependence, limited data for algorithm training [205,206], and the need for human experts selecting images before analysis [205,207,209].

#### 3.3.4. Pelvic Floor

The assessment of pelvic floor dysfunction is a highly essential and sensitive topic in gynecological examination due to its consequences on women’s health-related quality of life. Transvaginal US is the preferred diagnostic method, enabling the assessment of pelvic organ integrity, dynamic of pelvic floor function during Valsalva maneuver, and diagnosis of pelvic organ prolapse.

This review includes six articles on the introduced topic, with only one being designed as a prospective, randomized-controlled clinical trial [210]. Publication years ranged from 2019 to 2023. Two studies used 3D US images [211,212], two 2D [213,214,215], two of them derived 2D images from a 3D/4D data set [214,215], and one did not specify the type of image [210].

The assessment of the pelvic floor muscles and measurement of pelvic anatomical landmarks were addressed in all studies, while two focused on the diagnosis of pelvic organ prolapse [212,213]. Reliable automated plane detection and measurements were obtained results in all of the studies. Three studies were able to show the significantly reduced time between manual and automatic image evaluation, from up to 15 minutes to 1.27 seconds [211,212,213], concluding in saved clinician’s time for better bedside patient care. Limiting aspects encompassed high operator dependency [211,212], homogeneity of data when exclusively using cases of affected women [212,213], and the need for manual selection of ROI before AI image processing [211,213,214].

#### 3.3.5. Other Applications in Gynecology

Further fields of applications were found in the process of this literature review. In total, 11 articles were summarized in this section, including the topics of endometriosis [216,217], premature ovarian failure [218,219], uterine fibroids [220,221], follicle tracking [222,223], and ectopic pregnancies [224,225]. Another study addressed the issue of poor image quality in 3D US images due to data processing and showed that AI image enhancement methods could produce increased 3D image quality with user-preferential flexibility in both gynecological and obstetric US images [226]. The retrospective study of Huo et al. showed that AI-assisted US improved the accuracy of uterine fibroid assessment of young sonographers, but, summarized that AI applications rather assist than replace human observers [50].

##### Endometriosis

Two included articles discussed the sensitive topic of endometriosis, which can be problematic for both physician and patient due to complex clinical management and impaired quality of life in affected women [216,217]. Both studies had the usage of transvaginal 2D US videos and the missing histopathological or surgical confirmation in common, but focused on two different manifestations of endometriosis. Maicas et al. developed a highly accurate AI model for the classification of the pouch of Douglas obliteration as a cause of pelvic inflammation often seen in endometriosis via detection of the so-called ‘sliding sign’ [216]. In comparison, the results of Raimondo et al. showed a low sensitivity of the AI model to detect adenomyosis, but a high specificity, interpreted as a useful tool to rather exclude than detect adenomyosis [217].

##### Uterine Fibroids

In two of the included studies, the automated detection of uterine fibroids was analyzed. The retrospective study of Huo et al. showed that AI-assisted US improved the accuracy of uterine fibroid assessment of young sonographers, but, summarized that AI applications rather assist than replace human observers [220]. Yang et al. proposed an AI algorithm for the detection of fibroids, which facilitated pre-operative guidance and interventional therapy [221].

##### Premature Ovarian Failure

Premature ovarian failure or insufficiency is defined by the interruption of ovarian function before the onset of menopause, affects around 1% in women aged 40, and can cause amenorrhea or infertility [227]. Beside anamnesis and laboratory results on the hormone level, transvaginal US is the primary diagnostic tool to assess ovarian characteristics. This review lists two studies on this topic, evidencing that ovarian artery flow parameters obtained by AI analyzed color Doppler imaging can be used as a predictive factor, and both AI models showed reliability for disease prediction [210,218].

##### Follicle Tracking

In reproductive medicine, the evaluation of follicles after ovarian stimulation or the functional ovarian reserve in patients suffering from infertility is an important diagnostic component performed via US. Two included studies, of which one had a prospective, randomized-controlled design, showed increased accuracy of follicle evaluation and reduced examination time by using AI-assisted 2D and 3D US [222,223]. The mentioned limitations included cost-intensified AI-assisted machines and possible reduced image quality in obese patients [223].

##### Ectopic Pregnancy

In comparison with the use of AI in US for image analysis, two studies published an approach to use US images of ectopic pregnancies to build an ontology with a reference image collection for specific diagnostic signs (e.g., ‘ring of fire’). The prognosis of ectopic pregnancy is known to be dependent on the correctness and timing of diagnosis, for which the research groups Maurice et al. and Dhombres et al. showed that a knowledge base for US image annotations as a clinical decision support system based on this ontology significantly improved the timing of diagnosis [224,225].

## 4. Discussion

This systematic literature review presents an overview on applications for AI in US imaging in the medical field of OB/GYN. Relatively more publications were found to be suitable for inclusion that focused on applications in the field of obstetrics (148 versus 41 studies), possibly due to the predominance of US indications in this field. US is the preferred imaging method during pregnancy for fetal and maternal disorders for its low radiation exposure and possibility of real-time examination. In contrast with that, gynecological disorders such as different cancer entities and pelvis-related diseases benefit from other imaging methods such as MRI or CT. In the current literature, not only US, but also MRI applications profit from AI assistance, for example in fetal lung texture analysis [228,229] or cervical cancer diagnosis [230]. In the following, the benefits and limitations of AI application in OB/GYN US imaging are summarized.

### 4.1. Benefits

In general, AI in US imaging has the potential to reduce inter- and intra-observer variability by automating processes of image acquisition and interpretation [8]. AI-assisted US is able to significantly reduce examination time, showing decreased image acquisition times from minutes to seconds [211,212], thus, minimizing clinician’s workload [33] and enabling the sonographer to focus on the interpretation of the obtained images [140]. These advantages are of the utmost importance, especially in the clinical setting and in times of shortage of experienced health care personnel. In addition to this, AI models have been designed for image acquisition and classification, but also for facilitating or omitting repetitive work-intense tasks such as scan report production or captioning of US videos [140,159].

Not only clinicians profit from AI usage in US, but also patients, as AI helps to improve diagnostic accuracy and provides diagnostic safety. For example, the use of AI-assisted US has been shown to reduce the amount of unnecessary hospital admissions due to misdiagnosis and unnecessary breast biopsies [197,199,200]. This fact may reduce not only heath care costs, but, more importantly, diminish psychological burden for patients with unsecure diagnosis fearing the need for further diagnostic and intervention in inconclusive imaging results. In this context, AI in clinical settings can positively impact an individual patients’ life. Another example of direct patient benefit is the finding that in high-risk patients with ectopic pregnancies, reduced timing of diagnosis may result in an improved outcome [224]. AI models can also help to increase diagnostic accuracy, for example when US image quality is impeded by a thickened abdominal wall in obese patients [180]. Moreover, the advantages in pre-operative risk stratification or intraoperative assistance are described in both subspecialties of OB/GYN, e.g., in pre-operative endometrial cancer staging [209] and for fetoscopic surgical interventions [69,127]. Because of its reduction in examination time, AI-assisted US also has the potential to allow for cost-effective, population-based screening methods, e.g., for breast US [193,198,199]. Remarkably, when contextual clinical information is additionally incorporated in the AI model, the level of misclassification and misdiagnosis has been shown to be reduced [23,26]. To sum up, AI is not only a technical advantage when focusing on the imaging quality and accuracy, but, even more importantly, there is a clear benefit for an individual patient’s health care.

Nevertheless, AI-assisted US also helps to improve clinical education, which is well known to be neglected by a shortage of experienced clinicians and increased workload, especially in the recent pandemic times. It can support US novices in skill training and enables non-experts the acquisition of US images [160], e.g., for telehealth approaches in times of shortage of expert sonographers [50]. It, therefore, is of public health relevance, by reducing costs and the need for sonography experts [35,39]. In this framework, AI models are additionally able to enhance image acquisition and diagnostic accuracy in point-of-care US devices, which is of particular significance for application in low-resource settings and medically underserved areas [34,145,148,199].

### 4.2. Limitations

The main limitation of AI models in US imaging described in the summarized literature was the fact that most AI models still need experts for image acquisition, image or ROI selection to obtain an adequate image quality for accurate model performance [6,205,209,216], and for interpretation of the results [51,220]. In applications of tissue analysis such as assessment of the endometrium in gynecology [207,209] and fetal lung texture [162], or identification of the cervix [166] in obstetrics, manual selection of the ROI is still a limiting aspect in AI performance. These findings are in accordance with the often noted statement that AI models are primarily intended to assist the clinician, not to replace them [6,231]. This limitation is of major importance to discuss as it underlines the requirement for humans in performing, analyzing, supervising, interpreting, and taking clinical consequences of AI produced results.

The irreplaceable need for experts will be understandable when working out other limitations of AI usage. In pattern recognition tasks, some AI models can fail when subtle differences are diagnosis-relevant, e.g., in borderline findings or in small regions of interest such as endometrial thickness or fetal brain structures [95,201,208]. A change in US probe or modality may also lead to misclassification, e.g., when comparing abdominal or vaginal US images [185]. Furthermore, AI model performance in 2D and 3D US imaging can be limited due to imaging artefacts and noise, especially when automated tissue analysis is intended, for example, for fetal lung assessment, whereas the method of MRI for this specific application seems future-oriented and promising [127,163]. In the clinical setting, the assessment of fetal lungs in terms of texture and volume can be relevant for prenatal diagnosis, risk classification, prediction of prognosis, and therapy planning in fetal congenital diaphragmatic hernia, profiting from the combination of the imaging modalities of US and MRI [228,232].

In obstetric US, AI models designed for automatic biometric measurements are usually restraint to a specific range of GA and can fail in images of different GA [34,40,78]. Small structures such as the fetal limbs are prone to failing AI recognition [26,138,140], as well as the differentiation of structures within similar tissue textures [113]. Furthermore, real-time application is of particular importance in obstetric US and some authors noted the missing possibility for real-time application of various AI models, interestingly affecting especially those that are designed for intrapartum application [42,127,172,173,174]. This limitation may be due to the great use of computational power and memory of AI algorithms. One leading limitation of AI algorithms in obstetric US is the dependence on fetal position and movement. In fetal echocardiography in particular, most presented AI models have been trained with apical 4CV, ignoring the reality of heterogenous US images obtained from different scanning angles in clinical routine [57]. As a solution to this issue and an emerging research focus, the detection of the fetal heart as a ROI in US images can be performed by AI models [72,73].

However, not only the AI models itself, but also the study designs for model development and analysis summarized in this review bear some limitations that have an influence on model development and performance. As AI algorithms are usually dependent on large data sets for training, the detection of rare pathologies is limited due to missing training of pattern recognition models [132,133]. As most of the studies are performed with data obtained from healthy subjects or healthy fetuses, miscalculation or misdiagnosis may occur in case of pathologies [35,95,145]. Other factors based on study design that influence model performance are single study center, single observer or sonographer, single US device, small sample sizes, missing long-term data, and missing clinical validation.

Nevertheless, as perfectly outlined in the state-of-the-art review by Drukker et al., the clinical applicability of AI algorithms is still limited due to fears and concerns of clinicians regarding the safety or stability of the algorithms, trustworthiness, ethical background, privacy, and professional liability [3]. Where there is research about AI, it is also indispensable to mention ethical aspects of its application. The World Health Organization guidance for the ethics and governance of artificial intelligence for health states that it “recognizes that AI holds great promise for the practice of public health and medicine” [233], but also stresses the important aspect of ethical challenges, which must be addressed due to the fast-developing technologies. Drukker et al. stressed the importance of a better interdisciplinary research on AI applications of technicians and clinicians to reduce the difficulties and insecurities of clinicians when facing the complex methods of AI systems resulting in missing trust in these systems [3]. This aspect is particularly addressed by the concept of ‘explainable AI’, which is used in the studies of Sakai et al. and Dong et al. [7,201]. To sum up, limitations in the applications of AI algorithms are abundant, especially because most study settings seem inadequate for the evaluation of clinical applicability. Considering the fact that the technique of AI and its emerging systems is relatively new in the medical field, it is comprehensible that clinical approved results are missing.

### 4.3. Strengths and Limitations of This Review

One important advantage of this review is the inclusion of a reasonable number of publications over an extensive period of time, with no restrictions regarding year of publication. The included literature is categorized among their subspecialty and research topics, allowing for a visualized overview of the current research interest on the one hand, as well as an idea of still underrepresented tasks for AI applications in further research on the other hand.

Regarding the distribution of literature among the subspecialties of OB/GYN, one limiting aspect of this study may be the search query containing the extra keyword ‘pregnancy’, which is likely to have influenced a discrepancy in the obtained records in favor of obstetric studies. Another important limiting aspect might be the fact that this review includes research articles from engineering literature, which are known to have a technical viewpoint and fail to assess clinical applicability. Most applications of AI US imaging in these technical articles are still experimental and preliminary work and have not been sufficiently assessed for clinical applicability, which was also stated in the review of Dhombres et al. [234]. As these technical studies are developed by engineers, they are difficult to understand for clinicians, bringing up the discussion about the urgent need for an improved interface between AI specialists and clinicians applying AI technology in real-life scenarios [231]. Lastly, a classification of the presented AI applications in the technologic subcategories of regression modeling, population classification, and image segmentation would be of further interest and should be considered for further research. In the realm of regression modeling, AI algorithms can predict crucial parameters, such as fetal growth, aiding clinicians in identifying potential complications early on. Moreover, AI-driven classification systems can enhance the accuracy of diagnoses, ensuring a higher level of precision in identifying abnormalities or diseases. The segmentation applications of AI can assess the way organs and structures are delineated in ultrasound images, offering accuracy in complex anatomical analyses.

## 5. Conclusions

Applications for AI-assisted US widely range from fetal biometry, echocardiography, neurosonography, or the estimation of gestational age in obstetrics, to the identification of adnexal or breast masses and the assessment of endometrium or pelvic floor in gynecology. The applications for AI-assisted US in OB/GYN are especially numerous in the subspecialty of obstetrics, where the imaging method of US is of particular significance. However, while most studies are of technical nature and studies are designed by AI engineers, most of presented literature lack clinical applicability. This systematic literature review displays the variety of research topics on AI applications in US imaging in OB/GYN, including sparsely represented and potentially emerging topics for further research.

In conclusion, with abundant evidence, we can pronounce to live and evolve the era of 5D ultrasound, as AI algorithms add and will add a momentous further dimension to the existing US imaging methods in OB/GYN.

## Figures and Tables

**Table 1:** PICOS search tool headings for literature evaluation [12].

PICOS Search Tool Headings for Literature Evaluation	
Participants	Examiner: Healthcare professionals in OB/GYN or radiology, AI specialists Patients: Healthy pregnant and non-pregnant women or women with any gynecological or obstetric disease/complication, OB/GYN training models
Intervention or Exposure	AI-assisted US applications
Comparison	Comparison of AI US algorithms to human US examiners or another AI algorithm
Outcome	Fields of AI applications in OB/GYN US imaging, benefits and limitations of AI usage, future aspects for emerging fields of applications
Study type	Published literature of any design, excluding trial protocols and reviews

## Data Availability

The data presented in this study are available in the article or Supplementary Material here.

## Supplementary Materials

The following supporting information can be downloaded at: https://www.mdpi.com/article/10.3390/jcm12216833/s1, Table S1: Overview of included literature on artificial intelligence applications in ultrasound for the subspecialty of obstetrics; Table S2: Overview of included literature on artificial intelligence applications in ultrasound for the subspecialty of gynecology.

## Abbreviations

2/3/4/5D	Two/three/four/five-dimensional
4CV	Four-chamber view
AC	Abdominal circumference
AI	Artificial intelligence
CHD	Congenital heart disease
CRL	Crown-rump-length
CT	Computed tomography
FINE	Fetal intelligent navigation echocardiography
GA	Gestational age
HC	Head circumference
ISUOG	International Society of Ultrasound in Obstetrics & Gynecology
MRI	Magnetic resonance imaging
NT	Nuchal translucency
OB/GYN	Obstetrics and gynecology
ROI	Region of interest
US	Ultrasound
