# Supplementary material for: Evolving the Era of 5D Ultrasound? A Systematic Literature Review on the Applications for Artificial Intelligence Ultrasound Imaging in Obstetrics and Gynecology
Source: J Clin Med. 2023 Oct 29;12(21):6833. doi: 10.3390/jcm12216833 (PMC10649694; doi:10.3390/jcm12216833)
Supplement: Supplementary file 1 [file jcm-12-06833-s001.zip › jcm-2651594-supplementary.pdf]

Table S1: Overview of included literature on artificial intelligence applications in ultrasound for the subspecialty of obstetrics. US: Ultrasound.

Authors	Year	Titel	Study design	Type of US	Analyzed images/cases	Field of application
**Alansary et al.**	2019	Evaluating reinforcement learning agents for anatomical landmark detection.	Retrospective	2D	72 images	Fetal neurosonography
**Alsharid et al.**	2022	Gaze-assisted automatic captioning of fetal ultrasound videos using three-way multi-modal deep neural networks.	Retrospective	2D videos	10 videos	Workflow
**Alzubaidi et al.**	2022	Ensemble Transfer Learning for Fetal Head Analysis: From Segmentation to Gestational Age and Weight Prediction.	Retrospective	2D	1,334 images from 551 women	Gestational age
**Ambroise Grandjean et al.**	2018	Artificial intelligence assistance for fetal head biometry: Assessment of automated measurement software.	Prospective, cross-sectional	2D/3D	30 patients	Fetal biometry
**Andreasen et al.**	2023	Multi-centre deep learning for placenta segmentation in obstetric ultrasound with multi-observer and cross-country generalization.	Prospective, multi-center	2D	7,500 images	Placenta
**Arnaout et al.**	2021	An ensemble of neural networks provides expert-level prenatal detection of complex congenital heart disease.	Retrospective, multi-center	2D	107,823 images from 1,326 cases	Fetal echocardiography
**Arroyo et al.**	2022	No sonographer, no radiologist: New system for automatic prenatal detection of fetal biometry, fetal presentation, and placental location.	Retrospective	2D	58 women	Fetal biometry
**Asadpour et al.**	2023	Automated placental abruption identification using semantic segmentation, quantitative features, SVM, ensemble and multi-path CNN.	Retrospective	2D	645 images	Placenta
**Bai et al.**	2022	A framework for computing angle of progression from transperineal ultrasound images for evaluating fetal head descent using a novel double branch network.	Retrospective	2D	4,013 images	Intrapartum sonography
**Baumgartner et al.**	2017	SonoNet: Real-Time Detection and Localisation of Fetal Standard Scan Planes in Freehand Ultrasound.	Retrospective	2D videos	33,599 images, 2,638 videos	Fetal biometry
**Baykal et al.**	1994	Interpretation of Doppler blood flow velocity waveforms using neural networks.	Prospective	Doppler	600 datasets from 199 pregnancies	Placenta
**Beksaç et al.**	1996	An intelligent diagnostic system for the assessment of gestational age based on ultrasonic fetal head measurements.	Retrospective	2D	613 images from 143 fetuses	Gestational age
**Beksaç et al.**	1996	A computerized diagnostic system for the interpretation of umbilical artery blood flow velocity waveforms.	Prospective	Doppler	1,090 from 194 pregnancies	Placenta
**Beksaç et al.**	1996	An automated intelligent diagnostic system for the interpretation of umbilical artery Doppler velocimetry.	Prospective	Doppler	668 images from 254 women	Placenta
**Bonet-Carne et al.**	2015	Quantitative ultrasound texture analysis of fetal lungs to predict neonatal respiratory morbidity.	Prospective	2D images	900 images	Fetal lung maturation

**Burgos-Artizzu et al.**	2020	Evaluation of deep convolutional neural networks for automatic classification of common maternal fetal ultrasound planes.	Prospective	2D	12,400 images from 1,792 patients	Fetal biometry
**Burgos-Artizzu et al.**	2021	Analysis of maturation features in fetal brain ultrasound via artificial intelligence for the estimation of gestational age.	Prospective	2D	8,580 images from 2,034 patients	Fetal neurosonography
**Carneiro et al.**	2007	Automatic fetal measurements in ultrasound using constrained probabilistic boosting tree.	Retrospective	2D	3,887 images	Fetal biometry
**Carneiro et al.**	2008	Detection and measurement of fetal anatomies from ultrasound images using a constrained probabilistic boosting tree.	Retrospective	2D	4,759 images	Fetal biometry
**Cengizler et al.**	2020	A Nature-Inspired Search Space Reduction Technique for Spine Identification on Ultrasound Samples of Spina Bifida Cases.	Retrospective	2D	14 patients	Fetal malformations
**Chen et al.**	2017	Ultrasound Standard Plane Detection Using a Composite Neural Network Framework.	Retrospective	2D videos	50,634 images from 1,231 videos	Fetal biometry
**Chen et al.**	2015	Standard Plane Localization in Fetal Ultrasound via Domain Transferred Deep Neural Networks.	Retrospective	2D videos	20,660 images from 519 videos	Fetal biometry
**Chen et al.**	2020	A preliminary study to quantitatively evaluate the development of maturation degree for fetal lung based on transfer learning deep model from ultrasound images.	Retrospective	2D	332 patients	Fetal lung maturation
**Cho et al.**	2021	Automated ultrasound assessment of amniotic fluid index using deep learning.	Retrospective	2D	435 images from 255 patients	Miscellaneous
**Compagnone et al.**	2022	Artificial intelligence enhanced ultrasound (AI-US) in a severe obese parturient: a case report.	Case Report	2D	1 case	Miscellaneous
**Dan et al.**	2023	DeepGA for automatically estimating fetal gestational age through ultrasound imaging.	Retrospective	2D	10,413 images from 7,113 subjects	Gestational age
**Deng et al.**	2012	A hierarchical model for automatic nuchal translucency detection from ultrasound images.	Retrospective	2D	690 images	Fetal malformations
**Di Vece et al.**	2022	Deep learning-based plane pose regression in obstetric ultrasound.	Retrospective	2D/3D	6 volumes from 1 phantom	Fetal neurosonography
**Dong et al.**	2020	A Generic Quality Control Framework for Fetal Ultrasound Cardiac Four-Chamber Planes.	Retrospective	2D	7,032 images	Fetal echocardiography
**Dozen et al.**	2020	Image Segmentation of the Ventricular Septum in Fetal Cardiac Ultrasound Videos Based on Deep Learning Using Time-Series Information.	Prospective	2D videos	421 videos from 211 women	Fetal echocardiography
**Drukker et al.**	2021	Transforming obstetric ultrasound into data science using eye tracking, voice recording, transducer motion and ultrasound video.	Prospective	2D/3D, Doppler	1,158,782 videos from 341 videos	Workflow
**Drukker et al.**	2022	Clinical workflow of sonographers performing fetal anomaly ultrasound scans: deep-learning-based analysis.	Prospective	2D/3D/4D, Doppler	496 scans	Workflow

**Du et al.**	2021	Application of ultrasound-based radiomics technology in fetal-lung-texture analysis in pregnancies complicated by gestational diabetes and/or pre-eclampsia.	Retrospective	2D	430 images from 548 women	Fetal lung maturation
**Du et al.**	2022	Ultrasound-based radiomics technology in fetal lung texture analysis prediction of neonatal respiratory morbidity.	Retrospective	2D images	295 images	Fetal lung maturation
**Emery et al.**	2007	Computer-assisted navigation applied to fetal cardiac intervention.	Prospective	2D/3D	10 pregnant sheep	Fetal echocardiography
**Gembicki et al.**	2020	Semiautomatic Fetal Intelligent Navigation Echocardiography Has the Potential to Aid Cardiac Evaluations Even in Less Experienced Hands.	Prospective	2D/4D	30 fetuses	Fetal echocardiography
**Ghi et al.**	2022	Novel artificial intelligence approach for automatic differentiation of fetal occiput anterior and non-occiput anterior positions during labor.	Prospective, multi-center	2D	1,219 images from 1,219 women	Intrapartum sonography
**Gofer et al.**	2022	Machine Learning Algorithms for Classification of First-Trimester Fetal Brain Ultrasound Images.	Retrospective	2D	80 images from 56 fetuses	Fetal neurosonography
**Gomes et al.**	2022	A mobile-optimized artificial intelligence system for gestational age and fetal malpresentation assessment.	Retrospective	2D videos	521 patients	Gestational age
**Gong et al.**	2020	Fetal Congenital Heart Disease Echocardiogram Screening Based on DGACNN: Adversarial One-Class Classification Combined with Video Transfer Learning.	Retrospective	2D	not specified	Fetal echocardiography
**Gupta et al.**	2011	Segmentation of 2D fetal ultrasound images by exploiting context information using conditional random fields.	Retrospective	2D	2 images	Image quality
**Gupta et al.**	2022	Ultrasound placental image texture analysis using artificial intelligence to predict hypertension in pregnancy.	Prospective	2D, color Doppler	429 cases	Placenta
**Han et al.**	2022	Adoption of Compound Echocardiography under Artificial Intelligence Algorithm in Fetal Congenial Heart Disease Screening during Gestation.	Retrospective	2D, coler Doppler	9,654 fetuses	Fetal echocardiography
**He et al.**	2022	B-Ultrasound Image Analysis of Intrauterine Pregnancy Residues after Mid-Term Pregnancy Based on Smart Medical Big Data.	Retrospective	2D	106 patients	Maternal factors
**Herling et al.**	2021	Automated quantitative evaluation of fetal atrioventricular annular plane systolic excursion.	Retrospective	2D videos, color Doppler	343 fetuses	Fetal echocardiography
**Hesse et al.**	2022	Subcortical segmentation of the fetal brain in 3D ultrasound using deep learning.	Retrospective	3D	537 images	Fetal neurosonography
**Hu et al.**	2019	Automated Placenta Segmentation with a Convolutional Neural Network Weighted by Acoustic Shadow Detection.	Prospective	2D	1,364 images from 247 patients	Placenta
**Huang et al.**	2018	VP-Nets : Efficient automatic localization of key brain structures in 3D fetal neurosonography.	Retrospective	3D	285 scans	Fetal neurosonography
**Jang et al.**	2018	Automatic Estimation of Fetal Abdominal Circumference From Ultrasound Images.	Retrospective	2D	13,261 images from 88 cases	Fetal biometry
**Kaplan et al.**	2022	PFP-LHCINCA: Pyramidal Fixed-Size Patch-Based Feature Extraction and Chi-Square Iterative Neighborhood Component Analysis for Automated Fetal Sex Classification on Ultrasound Images.	Retrospective	2D	671 women	Miscellaneous
**Kim et al.**	2018	Machine-learning-based automatic identification of fetal abdominal circumference from ultrasound images.	Retrospective	2D	174 images	Fetal biometry

**Lee et al.**	2023	Development of a Machine Learning Model for Sonographic Assessment of Gestational Age.	Retrospective	2D images/videos	3,842 patients	Gestational age
**Lee et al.**	2023	Machine learning for accurate estimation of fetal gestational age based on ultrasound images.	Retrospective	2D images	293,811 images from 4233 pregnancies	Gestational age
**Lei et al.**	2015	Discriminative Learning for Automatic Staging of Placental Maturity via Multi-layer Fisher Vector.	Retrospective	2D	443 images	Placenta
**Li et al.**	2018	Automatic Fetal Head Circumference Measurement in Ultrasound Using Random Forest and Fast Ellipse Fitting.	Retrospective	2D	669 images	Fetal biometry
**Li et al.**	2020	Automated measurement network for accurate segmentation and parameter modification in fetal head ultrasound images.	Retrospective	2D	1,334 images from 551 women	Fetal biometry
**Li et al.**	2014	Automatic staging of placental maturity based on dense descriptor.	Retrospective	2D	311 images	Placenta
**Lin et al.**	2022	How much can AI see in early pregnancy: A multi-center study of fetus head characterization in week 10-14 in ultrasound using deep learning.	Retrospective	2D	1,528 images from 1,519 women	Fetal neurosonography
**Lin et al.**	2019	Multi-task learning for quality assessment of fetal head ultrasound images.	Retrospective	2D	1,771 images	Fetal neurosonography
**Lin et al.**	2022	Use of real-time artificial intelligence in detection of abnormal image patterns in standard sonographic reference planes in screening for fetal intracranial malformations.	Retrospective	2D images/videos	43,890 images from 16,297 pregnancies, 169 videos from 166 pregnancies	Fetal neurosonography
**Liu et al.**	2021	Doppler Ultrasound Imaging Combined with Fetal Heart Detection in Predicting Fetal Distress in Pregnancy-Induced Hypertension under the Guidance of Artificial Intelligence Algorithm.	Retrospective	Doppler	120 women	Maternal factors
**Looney et al.**	2021	Fully Automated 3-D Ultrasound Segmentation of the Placenta, Amniotic Fluid, and Fetus for Early Pregnancy Assessment.	Retrospective	3D	2,393 volumes	Early pregnancy
**Looney et al.**	2018	Fully automated, real-time 3D ultrasound segmentation to estimate first trimester placental volume using deep learning.	Retrospective	3D	2,393 patients	Placenta
**Lu et al.**	2022	Multitask Deep Neural Network for the Fully Automatic Measurement of the Angle of Progression.	Retrospective	2D	1,964 images from 104 women	Intrapartum sonography
**Luo et al.**	2021	A Prenatal Ultrasound Scanning Approach: One-Touch Technique in Second and Third Trimesters.	Prospective	2D	1,005 women	Fetal biometry
**Ma et al.**	2020	Diagnostic performance of fetal intelligent navigation echocardiography (FINE) in fetuses with double-outlet right ventricle (DORV).	Prospective	3D/4D	30 fetuses	Fetal echocardiography
**Maraci et al.**	2017	A framework for analysis of linear ultrasound videos to detect fetal presentation and heartbeat.	Retrospective	2D videos	323 videos	Early Pregnancy
**Maraci et al.**	2020	Toward point-of-care ultrasound estimation of fetal gestational age from the trans-cerebellar diameter using CNN-based ultrasound image analysis.	Retrospective	2D images	8,736 images	Gestational age

**Matthew et al.**	2022	Exploring a new paradigm for the fetal anomaly ultrasound scan: Artificial intelligence in real time.	Prospective	2D	23 women	Fetal malformations
**Meenakshi et al.**	2019	Segmentation and Boundary Detection of Fetal Kidney Images in Second and Third Trimesters Using Kernel-Based Fuzzy Clustering.	Retrospective	2D	50 images	Fetal malformations
**Meng et al.**	2019	Weakly Supervised Estimation of Shadow Confidence Maps in Fetal Ultrasound Imaging.	Retrospective	2D	8,500 images from 2,694 patients	Image quality
**Miyagi et al.**	2022	Artificial intelligence to understand fluctuation of fetal brain activity by recognizing facial expressions.	Retrospective	4D	37 videos from 33 patients	Fetal face
**Miyagi et al.**	2021	Recognition of facial expression of fetuses by artificial intelligence (AI).	Retrospective	4D	147 images from 14 fetuses	Fetal face
**Namburete et al.**	2018	Fully-automated alignment of 3D fetal brain ultrasound to a canonical reference space using multi-task learning.	Retrospective	3D	739 volumes	Fetal neurosonography
**Namburete et al.**	2015	Learning-based prediction of gestational age from ultrasound images of the fetal brain.	Retrospective	3D	634 cases	Gestational age
**Ni et al.**	2014	Standard plane localization in ultrasound by radial component model and selective search.	Retrospective	2D videos	1,995 images, 223 videos	Fetal biometry
**Nurmaini et al.**	2021	Deep Learning-Based Computer-Aided Fetal Echocardiography: Application to Heart Standard View Segmentation for Congenital Heart Defects Detection.	Retrospective	2D	1,149 images from 100 women	Fetal echocardiography
**Nurmaini et al.**	2022	Deep Learning for Improving the Effectiveness of Routine Prenatal Screening for Major Congenital Heart Diseases.	Retrospective	2D videos	1,184 videos from 76 women	Fetal echocardiography
**Patra et al.**	2020	Hierarchical Class Incremental Learning of Anatomical Structures in Fetal Echocardiography Videos.	Retrospective	2D videos	not specified	Fetal echocardiography
**Pietrolucci et al.**	2023	Evaluation of an artificial intelligent algorithm (Heartassist™) to automatically assess the quality of second trimester cardiac views: a prospective study.	Prospective	2D	120 women	Fetal echocardiography
**Płotka et al.**	2022	Deep learning fetal ultrasound video model match human observers in biometric measurements.	Retrospective	2D images/videos	700 videos	Fetal biometry
**Pluym et al.**	2021	Accuracy of automated three-dimensional ultrasound imaging technique for fetal head biometry.	Prospective, observational	3D	143 women	Fetal biometry
**Pokaprakarn et al.**	2022	AI Estimation of Gestational Age from Blind Ultrasound Sweeps in Low-Resource Settings.	Prospective	2D images/videos	4,695 patients	Gestational age
**Pradipta et al.**	2022	Machine learning model for umbilical cord classification using combination coiling index and texture feature based on 2-D Doppler ultrasound images.	Retrospective	2D, Doppler	151 images	Placenta
**Prieto et al.**	2021	An automated framework for image classification and segmentation of fetal ultrasound images for gestational age estimation.	Retrospective, prospective	2D images	155,088 images from 8,843 studies	Gestational age

**Qi et al.**	2018	Automatic Lacunae Localization in Placental Ultrasound Images via Layer Aggregation.	Retrospective	2D	3,440 images from 34 scans	Placenta
**Qi et al.**	2017	Weakly Supervised Learning of Placental Ultrasound Images with Residual Networks.	Retrospective	2D	10,808 images from 60 volumes	Placenta
**Qiao et al.**	2023	A Pseudo-Siamese Feature Fusion Generative Adversarial Network for Synthesizing High-Quality Fetal Four-Chamber Views.	Retrospective	2D	not specified	Fetal echocardiography
**Rahman et al.**	2023	Demystifying evidential Dempster Shafer-based CNN architecture for fetal plane detection from 2D ultrasound images leveraging fuzzy-contrast enhancement and explainable AI.	Retrospective	2D	12,400 images	Fetal biometry
**Rueda et al.**	2015	Feature-based fuzzy connectedness segmentation of ultrasound images with an object completion step.	Retrospective	2D	81 images	Miscellaneous
**Ryou et al.**	2019	Automated 3D ultrasound image analysis for first trimester assessment of fetal health.	Retrospective	3D	65 volumes	Fetal malformations
**Sahli et al.**	2019	Supervised classification approach of biometric measures for automatic fetal defect screening in head ultrasound images.	Retrospective	2D	86 women	Fetal neurosonography
**Sakai et al.**	2022	Medical Professional Enhancement Using Explainable Artificial Intelligence in Fetal Cardiac Ultrasound Screening.	Retrospective	2D videos	334 videos from 160 cases	Fetal echocardiography
**Scharf et al.**	2023	How Automated Techniques Ease Functional Assessment of the Fetal Heart: Applicability of MPI+,Ñ¢ for Direct Quantification of the Modified Myocardial Performance Index.	Retrospective	2d videos, Doppler	85 fetuses	Fetal echocardiography
**Schilpzand et al.**	2022	Automatic Placenta Localization From Ultrasound Imaging in a Resource-Limited Setting Using a Predefined Ultrasound Acquisition Protocol and Deep Learning.	Prospective	2D	280,6574 images	Placenta
**Schwartz et al.**	2022	Fully Automated Placental Volume Quantification From 3D Ultrasound for Prediction of Small-for-Gestational-Age Infants.	Retrospective	2D/3D	124 images from 422 patients	Placenta
**Sciortino et al.**	2017	Automatic detection and measurement of nuchal translucency.	Retrospective	2D	382 images from 12 patients	Fetal malformations
**Sendra-Balcells et al.**	2023	Generalisability of fetal ultrasound deep learning models to low-resource imaging settings in five African countries.	Prospective	2D	12,454 images from 2925 patients	Fetal biometry
**Sharma et al.**	2021	Multi-Modal Learning from Video, Eye Tracking, and Pupillometry for Operator Skill Characterization in Clinical Fetal Ultrasound.	Retrospective	2D videos	2,309 videos from 370 scans	Workflow
**Sharma et al.**	2021	Knowledge representation and learning of operator clinical workflow from full-length routine fetal ultrasound scan videos.	Prospective	2D/3D/4D, Doppler	341 videos	Workflow
**Shozu et al.**	2020	Model-Agnostic Method for Thoracic Wall Segmentation in Fetal Ultrasound Videos.	Retrospective	2D	538 images, 280 videos from 256 cases	Fetal malformations
**Skelton et al.**	2021	Towards automated extraction of 2D standard fetal head planes from 3D ultrasound acquisitions: A clinical evaluation and quality assessment comparison.	Retrospective	2D/3D	551 images from 91 scans	Fetal neurosonography

**Sreelakshmy et al.**	2022	An Automated Deep Learning Model for the Cerebellum Segmentation from Fetal Brain Images.	Retrospective	2D	740 images	Fetal neurosonography
**Sridar et al.**	2019	Decision Fusion-Based Fetal Ultrasound Image Plane Classification Using Convolutional Neural Networks.	Retrospective	2D	4,074 images	Fetal biometry
**Sun et al.**	2022	Model application to quantitatively evaluate placental features from ultrasound images with gestational diabetes.	Prospective	2D, color Doppler	718 images	Placenta
**Sun et al.**	2023	Multimodal fusion model for classifying placenta ultrasound imaging in pregnancies with hypertension disorders.	Prospective	2D, color Doppler	654 women	Placenta
**Sur et al.**	2010	A novel technique for the semi-automated measurement of embryo volume: an intraobserver reliability study.	Retrospective	2D/3D	72 women	Early pregnancy
**Tang et al.**	2023	The Two-Stage Ensemble Learning Model Based on Aggregated Facial Features in Screening for Fetal Genetic Diseases.	Prospective	3D	932 images from 667 pregnancies	Fetal face
**Torrents-Barrena et al.**	2019	TTTS-GPS: Patient-specific preoperative planning and simulation platform for twin-to-twin transfusion syndrome fetal surgery.	Retrospective	3D	60 volumes	Placenta
**Torrents-Barrena et al.**	2021	Assessment of Radiomics and Deep Learning for the Segmentation of Fetal and Maternal Anatomy in Magnetic Resonance Imaging and Ultrasound.	Retrospective	3D	not specified	Placenta
**Tsai et al.**	2020	Automatic Fetal Middle Sagittal Plane Detection in Ultrasound Using Generative Adversarial Network.	Prospective	3D	218 volumes	Fetal malformations
**Van den Heuvel et al.**	2019	Automated Fetal Head Detection and Circumference Estimation from Free-Hand Ultrasound Sweeps Using Deep Learning in Resource-Limited Countries.	Prospective	2D	183 images	Fetal biometry
**Veronese et al.**	2023	Prenatal Diagnosis and Fetopsy Validation of Complete Atrioventricular Septal Defects Using the Fetal Intelligent Navigation Echocardiography Method.	Prospective	2D/4D	4 fetuses	Fetal echocardiography
**Walker et al.**	2022	Using deep-learning in fetal ultrasound analysis for diagnosis of cystic hygroma in the first trimester.	Retrospective	2D	289 images	Fetal malformations
**Wang et al.**	2022	Automated prediction of early spontaneous miscarriage based on the analyzing ultrasonographic gestational sac imaging by the convolutional neural network: a case-control and cohort study.	Retrospective/Prospective	2D	2,468 images from 1,234 women	Early pregnancy
**Wang et al.**	2022	Diagnosis of fetal total anomalous pulmonary venous connection based on the post-left atrium space ratio using artificial intelligence.	Retrospective	2D	319 fetuses	Fetal echocardiography
**Wang et al.**	2021	Recognition of Fetal Facial Ultrasound Standard Plane Based on Texture Feature Fusion.	Retrospective	2D	943 images	Fetal face
**Wang et al.**	2022	Value of Ultrasonic Image Features in Diagnosis of Perinatal Outcomes of Severe Preeclampsia on account of Deep Learning Algorithm.	Prospective	Doppler	280 women	Maternal factors
**Wang et al.**	2022	Task model-specific operator skill assessment in routine fetal ultrasound scanning.	Retrospective	2D videos	294 videos from 139 patients	Workflow
**Wu et al.**	2023	Application of Artificial Intelligence in Anatomical Structure Recognition of Standard Section of Fetal Heart.	Retrospective	2D	3,360 images	Fetal echocardiography
**Wu et al.**	2004	A novel algorithm for computer-assisted measurement of cervical length from transvaginal ultrasound images.	Retrospective	2D	101 images from 17 patients	Maternal factors

**Wu et al.**	2017	FUIQA: Fetal Ultrasound Image Quality Assessment With Deep Convolutional Networks.	Retrospective	2D	8,072 images from 492 videos	Image quality
**Xia et al.**	2021	Establish a normal fetal lung gestational age grading model and explore the potential value of deep learning algorithms in fetal lung maturity evaluation.	Retrospective	2D	7,013 images from 1,023 pregnancies	Fetal lung maturation
**Xie et al.**	2020	Using deep-learning algorithms to classify fetal brain ultrasound images as normal or abnormal.	Retrospective	2D/3D	29,419 images from 12,780 cases	Fetal neurosonography
**Xie et al.**	2020	Computer-aided diagnosis for fetal brain ultrasound images using deep convolutional neural networks.	Retrospective	2D images/videos	29,748 women	Fetal neurosonography
**Xu et al.**	2020	Simulating realistic fetal neurosonography images with appearance and growth change using cycle-consistent adversarial networks and an evaluation.	Retrospective	2D	not specified	Fetal neurosonography
**Xu et al.**	2020	DW-Net: A cascaded convolutional neural network for apical four-chamber view segmentation in fetal echocardiography.	Retrospective	2D	895 images	Fetal echocardiography
**Yang et al.**	2022	A new approach to automatic measure fetal head circumference in ultrasound images using convolutional neural networks.	Retrospective	2D	1,334 images from 551 women	Fetal biometry
**Yang et al.**	2021	Agent With Warm Start and Adaptive Dynamic Termination for Plane Localization in 3D Ultrasound.	Retrospective	3D	1,635 volumes	Fetal biometry
**Yang et al.**	2023	Classification of normal and abnormal fetal heart ultrasound images and identification of ventricular septal defects based on deep learning.	Retrospective	2D	not specified	Fetal echocardiography
**Yang et al.**	2022	Deep Learning Algorithm-Based Ultrasound Image Information in Diagnosis and Treatment of Pernicious Placenta Previa.	Prospective	Doppler	60 women	Placenta
**Yang et al.**	2019	Towards Automated Semantic Segmentation in Prenatal Volumetric Ultrasound.	Prospective	3D	104 women	Fetal malformations
**Yaqub et al.**	2017	A Deep Learning Solution for Automatic Fetal Neurosonographic Diagnostic Plane Verification Using Clinical Standard Constraints.	Retrospective	2D	19,838 images from 10,595 scans	Fetal neurosonography
**Yasrab et al.**	2023	A Machine Learning Method for Automated Description and Workflow Analysis of First Trimester Ultrasound Scans.	Prospective	2D videos	250 videos	Workflow
**Yasunari et al.**	2023	Fetal brain activity and the free energy principle.	Retrospective	4D	ns	Fetal face
**Yeo et al.**	2013	Fetal Intelligent Navigation Echocardiography (FINE): a novel method for rapid, simple, and automatic examination of the fetal heart.	Prospective	4D	51 datasets	Fetal echocardiography
**Yeung et al.**	2021	Learning to map 2D ultrasound images into 3D space with minimal human annotation.	Retrospective	2D/3D	195,000 images from 65 volumes	Fetal neurosonography
**Yin et al.**	2022	Evaluation of Nursing Effect of Pelvic Floor Rehabilitation Training on Pelvic Organ Prolapse in Postpartum Pregnant Women under Ultrasound Imaging with Artificial Intelligence Algorithm	Prospective, randomized-controlled	3D	60 patients	Image quality
**Yu et al.**	2022	A guiding approach of Ultrasound scan for accurately obtaining standard diagnostic planes of fetal brain malformation.	Prospective	2D/3D	3,200 images	Fetal neurosonography
**Yu et al.**	2017	Determination of Fetal Left Ventricular Volume Based on Two-Dimensional Echocardiography.	Retrospective	2D/3D	50 cases	Fetal echocardiography

**Yu et al.**	2018	A Deep Convolutional Neural Network-Based Framework for Automatic Fetal Facial Standard Plane Recognition.	Retrospective	2D	7,267 images	Fetal face
**Zeng et al.**	2021	Fetal Ultrasound Image Segmentation for Automatic Head Circumference Biometry Using Deeply Supervised Attention-Gated V-Net.	Retrospective	2D	1,354 images from 551 patients	Fetal biometry
**Zeng et al.**	2022	Efficient fetal ultrasound image segmentation for automatic head circumference measurement using a lightweight deep convolutional neural network.	Retrospective	2D	1,334 images from 551 women	Fetal biometry
**Zhang et al.**	2021	Automatic quality assessment for 2D fetal sonographic standard plane based on multitask learning.	Retrospective	2D	4,101 images	Fetal biometry
**Zhang et al.**	2022	Segmentation-Based vs. Regression-Based Biomarker Estimation: A Case Study of Fetus Head Circumference Assessment from Ultrasound Images.	Retrospective	2D	1,334 images from 551 women	Fetal biometry
**Zhang et al.**	2022	Development and Validation of a Deep Learning Model to Screen for Trisomy 21 During the First Trimester From Nuchal Ultrasonographic Images.	Retrospective	2D	822 women	Fetal malformations
**Zhao et al.**	2021	Visual-Assisted Probe Movement Guidance for Obstetric Ultrasound Scanning using Landmark Retrieval.	Prospective	3D	535,775 images	Workflow
**Zhu et al.**	2021	Automatic measurement of fetal femur length in ultrasound images: a comparison of random forest regression model and SegNet.	Retrospective	2D	436 images	Fetal biometry

Table S2: Overview of included literature on artificial intelligence applications in ultrasound for the subspecialty of gynecology. US: Ultrasound.

Authors	Year	Titel	Study design	Type of US	Analyzed images/cases	Field of application
**Al-Karawi et al.**	2021	An Evaluation of the Effectiveness of Image-based Texture Features Extracted from Static B-mode Ultrasound Images in Distinguishing between Benign and Malignant Ovarian Masses.	Retrospective	2D	242 images	Adnexal masses
**Amor et al.**	2009	Gynecologic imaging reporting and data system: a new proposal for classifying adnexal masses on the basis of sonographic findings.	Prospective	2D, color Doppler	187 masses from 171 women	Adnexal masses
**Aramendía-Vidaurreta et al.**	2016	Ultrasound Image Discrimination between Benign and Malignant Adnexal Masses Based on a Neural Network Approach.	Retrospective	3D	145 women	Adnexal masses
**Berg et al.**	2023	Toward AI-supported US Triage of Women with Palpable Breast Lumps in a Low-Resource Setting.	Prospective, multi-center	2D	758 masses from 300 women	Breast masses
**Browne et al.**	2023	AI: Can It Make a Difference to the Predictive Value of Ultrasound Breast Biopsy?	Retrospective	2D	403 cases	Breast masses
**Chen at al.**	2022	Deep Learning Prediction of Ovarian Malignancy at US Compared with O-RADS and Expert Assessment.	Retrospective	2D, color Doppler	422 women	Adnexal masses
**Chen et al.**	2023	Improving the Segmentation Accuracy of Ovarian-Tumor Ultrasound Images Using Image Inpainting.	Retrospective	2D	1,469 images	Adnexal masses

**Christiansen et al.**	2021	Ultrasound image analysis using deep neural networks for discriminating between benign and malignant ovarian tumors: comparison with expert subjective assessment.	Retrospective	2D, color Doppler	3,077 images from 758 women	Adnexal masses
**Dhombres et al.**	2017	Developing a knowledge base to support the annotation of ultrasound images of ectopic pregnancy.	Retrospective	not specified	208 images from 35 cases	Ectopic pregnancies
**Dong et al.**	2021	One step further into the blackbox: a pilot study of how to build more confidence around an AI-based decision system of breast nodule assessment in 2D ultrasound.	Retrospective	2D	785 images from 367 women	Breast masses
**Gao et al.**	2022	Deep learning-enabled pelvic ultrasound images for accurate diagnosis of ovarian cancer in China: a retrospective, multicentre, diagnostic study	Retrospective, multi-center	2D	592,275 images from 107,624 women	Adnexal masses
**Hsu et al.**	2022	Automatic ovarian tumors recognition system based on ensemble convolutional neural network with ultrasound imaging.	Prospective	2D	1,896 images from 587 women	Adnexal masses
**Huang et al.**	2022	Artificial intelligence breast ultrasound and handheld ultrasound in the BI-RADS categorization of breast lesions: A pilot head to head comparison study in screening program.	Prospective	2D videos	344 women	Breast masses
**Huh et al.**	2023	Tunable image quality control of 3-D ultrasound using switchable CycleGAN.	Prospective	3D	323 images	Image quality
**Huo et al.**	2023	Artificial intelligence-aided method to detect uterine fibroids in ultrasound images: a retrospective study.	Retrospective	2D	3,870 images from 667 women	Uterine fibroids
**Jung et al.**	2022	Ovarian tumor diagnosis using deep convolutional neural networks and a denoising convolutional autoencoder.	Retrospective	2D	1,613 images	Adnexal masses
**Liu et al.**	2022	Automatic Measurement of Endometrial Thickness From Transvaginal Ultrasound Images.	Retrospective	2D	8,119 images from 467 cases	Endometrium
**Magnuska et al.**	2022	Influence of the Computer-Aided Decision Support System Design on Ultrasound-Based Breast Cancer Classification.	Retrospective	2D	505 cases from 497 women	Breast masses
**Maicas at al.**	2021	Deep learning to diagnose pouch of Douglas obliteration with ultrasound sliding sign.	Prospective	2D videos	749 videos	Endometriosis
**Martínez Más et al.**	2019	Evaluation of machine learning methods with Fourier Transform features for classifying ovarian tumors based on ultrasound images.	Retrospective	2D	348 images	Adnexal masses
**Maurice et al.**	2017	Towards ontology-based decision support systems for complex ultrasound diagnosis in obstetrics and gynecology.	Retrospective	2D	206 images	Ectopic pregnancies
**Moro et al.**	2022	Developing and validating ultrasound-based radiomics models for predicting high-risk endometrial cancer	Retrospective, multi-center	2D	498 women	Endometrium
**Nero et al.**	2020	Germline BRCA 1-2 status prediction through ovarian ultrasound images radiogenomics: a hypothesis generating study (PROBE study).	Retrospective	2D	890 images from 255 women	Adnexal masses
**Noor et al.**	2020	Three-Dimensional Automated Volume Calculation (Sonography-Based Automated Volume Count) versus Two-Dimensional Manual Ultrasonography for Follicular Tracking and Oocyte Retrieval in Women Undergoing in vitro Fertilization-Embryo Transfer: a Randomized Controlled Trial	Prospective, randomized-controlled	2D/3D	130 women	Follicle tracking

**Park et al.**	2019	Endometrium segmentation on transvaginal ultrasound image using key-point discriminator.	Retrospective, multi-center	2D	3,372 images	Endometrium
**Pfob et al.**	2022	Intelligent multi-modal shear wave elastography to reduce unnecessary biopsies in breast cancer diagnosis (INSPiRED 002): a retrospective, international, multicentre analysis.	Retrospective, multi-center	2D	875 women	Breast masses
**Pfob et al.**	2022	The importance of multi-modal imaging and clinical information for humans and AI-based algorithms to classify breast masses (INSPiRED 003): an international, multicenter analysis	Prospective, multi-center	2D	1,288 women	Breast masses
**Raimondo et al.**	2023	Application of Deep Learning Model in the Sonographic Diagnosis of Uterine Adenomyosis.	Prospective	2D videos	100 videos from 100 women	Endometriosis
**Singh et al.**	2022	HaTU-Net: Harmonic Attention Network for Automated Ovarian Ultrasound Quantification in Assisted Pregnancy.	Retrospective	2D	197 women	Follicle tracking
**Szentimrey et al.**	2023	Automated segmentation and measurement of the female pelvic floor from the mid-sagittal plane of 3D ultrasound volumes.	Retrospective	3D	248 images from 135 women	Pelvic floor
**Van den Noort et al.**	2022	Automatic identification and segmentation of slice of minimal hiatal dimensions in transperineal ultrasound volumes.	Retrospective	2D	367 images from 116 women	Pelvic floor
**Van den Noort et al.**	2019	Deep learning enables automatic quantitative assessment of puborectalis muscle and urogenital hiatus in plane of minimal hiatal dimensions.	Retrospective	2D from 3D/4D	1,318 images	Pelvic floor
**Wang et al.**	2022	Automatic evaluation of endometrial receptivity in three-dimensional transvaginal ultrasound images based on 3D U-Net segmentation.	Retrospective	3D	85 women	Endometrium
**Williams et al.**	2021	Automatic Extraction of Hiatal Dimensions in 3-D Transperineal Pelvic Ultrasound Recordings.	Retrospective	3D	73 cases	Pelvic floor
**Wu et al.**	2022	Development and validation of a composite AI model for the diagnosis of levator ani muscle avulsion.	Retrospective	3D/4D	304 patients	Pelvic floor
**Yang et al.**	2023	Automatic Detection of Benign/Malignant Tumor in Breast Ultrasound Images using Optimal Features.	Retrospective	2D	1,680 images	Breast masses
**Yang et al.**	2023	Real-Time Automatic Assisted Detection of Uterine Fibroid in Ultrasound Images Using a Deep Learning Detector	Retrospective	2D	ns	Uterine fibroids
**Yu et al.**	2022	Diagnosis of Idiopathic Premature Ovarian Failure by Color Doppler Ultrasound under the Intelligent Segmentation Algorithm.	Retrospective	Color Doppler	120 women	Premature Ovarian failure
**Zhang et al.**	2022	Application of Transfer Learning and Feature Fusion Algorithms to Improve the Identification and Prediction Efficiency of Premature Ovarian Failure.	Prospective	2D/3D	100 women	Premature Ovarian failure
**Zhao et al.**	2023	Artificial intelligence diagnosis of intrauterine adhesion by 3D ultrasound imaging: a prospective study.	Prospective	3D	4,401 women	Endometrium
**Zhu et al.**	2022	Ultrasound Evaluation of Pelvic Floor Function after Transumbilical Laparoscopic Single-Site Total Hysterectomy Using Deep Learning Algorithm.	Prospective, randomized-controlled	not specified	3,619 images from 80 women	Pelvic floor
